# Multi-neuronal recording in unrestrained animals with all acousto-optic random-access line-scanning two-photon microscopy

**DOI:** 10.3389/fnins.2023.1135457

**Published:** 2023-06-14

**Authors:** Akihiro Yamaguchi, Rui Wu, Paul McNulty, Doycho Karagyozov, Mirna Mihovilovic Skanata, Marc Gershow

**Affiliations:** ^1^Department of Physics, New York University, New York, NY, United States; ^2^Center for Neural Science, New York University, New York, NY, United States; ^3^Neuroscience Institute, New York University, New York, NY, United States

**Keywords:** two-photon, drosophila, tracking microscopy, TAG lens, calcium imaging, acousto-optic, drosophila larva, motor system

## Abstract

To understand how neural activity encodes and coordinates behavior, it is desirable to record multi-neuronal activity in freely behaving animals. Imaging in unrestrained animals is challenging, especially for those, like larval *Drosophila melanogaster*, whose brains are deformed by body motion. A previously demonstrated two-photon tracking microscope recorded from individual neurons in freely crawling *Drosophila* larvae but faced limits in multi-neuronal recording. Here we demonstrate a new tracking microscope using acousto-optic deflectors (AODs) and an acoustic GRIN lens (TAG lens) to achieve axially resonant 2D random access scanning, sampling along arbitrarily located axial lines at a line rate of 70 kHz. With a tracking latency of 0.1 ms, this microscope recorded activities of various neurons in moving larval *Drosophila* CNS and VNC including premotor neurons, bilateral visual interneurons, and descending command neurons. This technique can be applied to the existing two-photon microscope to allow for fast 3D tracking and scanning.

## 1. Introduction

Calcium imaging is a versatile tool to monitor population neural activity with single-cell resolution; imaging in moving animals allows the study of the correlation between neural activity and behavior (Yang and Yuste, 2017). Two-photon (2P) imaging techniques image deeper tissues with less light scattering compared to methods that utilize linear (one photon) absorption processes (Helmchen and Denk, 2005). Although many 2P methods have been developed to record neural population activity (Lecoq et al., 2019; Grienberger et al., 2022) in immobilized animals, it has been particularly challenging to track and record activities from individual neurons during free behavior. These challenges are exacerbated in animals lacking a rigid skull-like enclosure, due to extensive motion-induced deformations.

The nematode *C. elegans* lacks a visual system, maintains rigidity through osmotic pressure, crawls smoothly on its side in a nearly planar path, and has been successfully studied using a range of fluorescence microscopy approaches (Clark et al., 2007; Faumont et al., 2011; Hendricks et al., 2012; Schrödel et al., 2013; Prevedel et al., 2014; Kato et al., 2015; Abrahamsson et al., 2016; Nguyen et al., 2016, 2017; Venkatachalam et al., 2016; Voleti et al., 2019; Nejatbakhsh et al., 2020). These techniques have not transferred to the *Drosophila* larva, whose peristaltic crawling induces 3D rotations, translations, and deformations of the brain that are out of sync with the animal's external movement (Sun and Heckscher, 2016). To address these issues, we previously developed a tracking microscope with two galvanometric mirrors and a tunable acoustic gradient (TAG) lens capable of tracking one or two neurons with closely spaced cell bodies (Karagyozov et al., 2018), but this method was not capable, during free behavior, of *in vivo* functional imaging from three or more neurons or from two widely spaced neurons. The principal obstacle was the loss of signal during the time it took inertia-limited mirrors to move the tracking spot from one neuron to the next.

Recent advances in 2P imaging have been made using acousto-optic deflectors (AODs) to enable high-speed random access scanning and to record neural activity in a variety of configurations (Lecoq et al., 2019). Two-photon microscopes employing two acousto-optic deflectors (AODs) allowed random access positioning in 2D (Iyer et al., 2006; Vučinić and Sejnowski, 2007; Otsu et al., 2008; Grewe et al., 2010; Jiang et al., 2012; Shao et al., 2012; Chamberland et al., 2017; Sakaki et al., 2020). These could be combined with an electrically tunable lens (ETL) to achieve faster axial scanning (Grewe et al., 2011), but the ~100 Hz bandwidth of an ETL is still too low for our tracking purposes. Acousto-optic lenses (AOL) that employ four AODs allow high-speed 3D random access two-photon imaging (Duemani Reddy et al., 2008; Kirkby et al., 2010; Konstantinou et al., 2016; Griffiths et al., 2020). The AOL is both expensive and dispersive due to the number and size of the AODs. Lensing is achieved by frequency chirping the driving elements, which have a limited bandwidth, so focusing far from the natural focus of the objective limits dwell time and cannot be achieved at the periphery of the field of view. In this paper, we describe an all acousto-optic 2P microscope combining two AODs for x-y deflection with a resonant lens for axial scanning, allowing for simplified optical alignment, higher throughput, and extended axial scan ranges at the edge of the field of view. We use this microscope to record the coordinated activities of multiple VNC neurons during forward locomotion, the activity of a descending central brain neuron correlated with the direction of locomotion it controls, and the light responses of visual interneurons located in opposite brain hemispheres.

## 2. Results

### 2.1. A random access line scanning microscope

**Figure 1:**
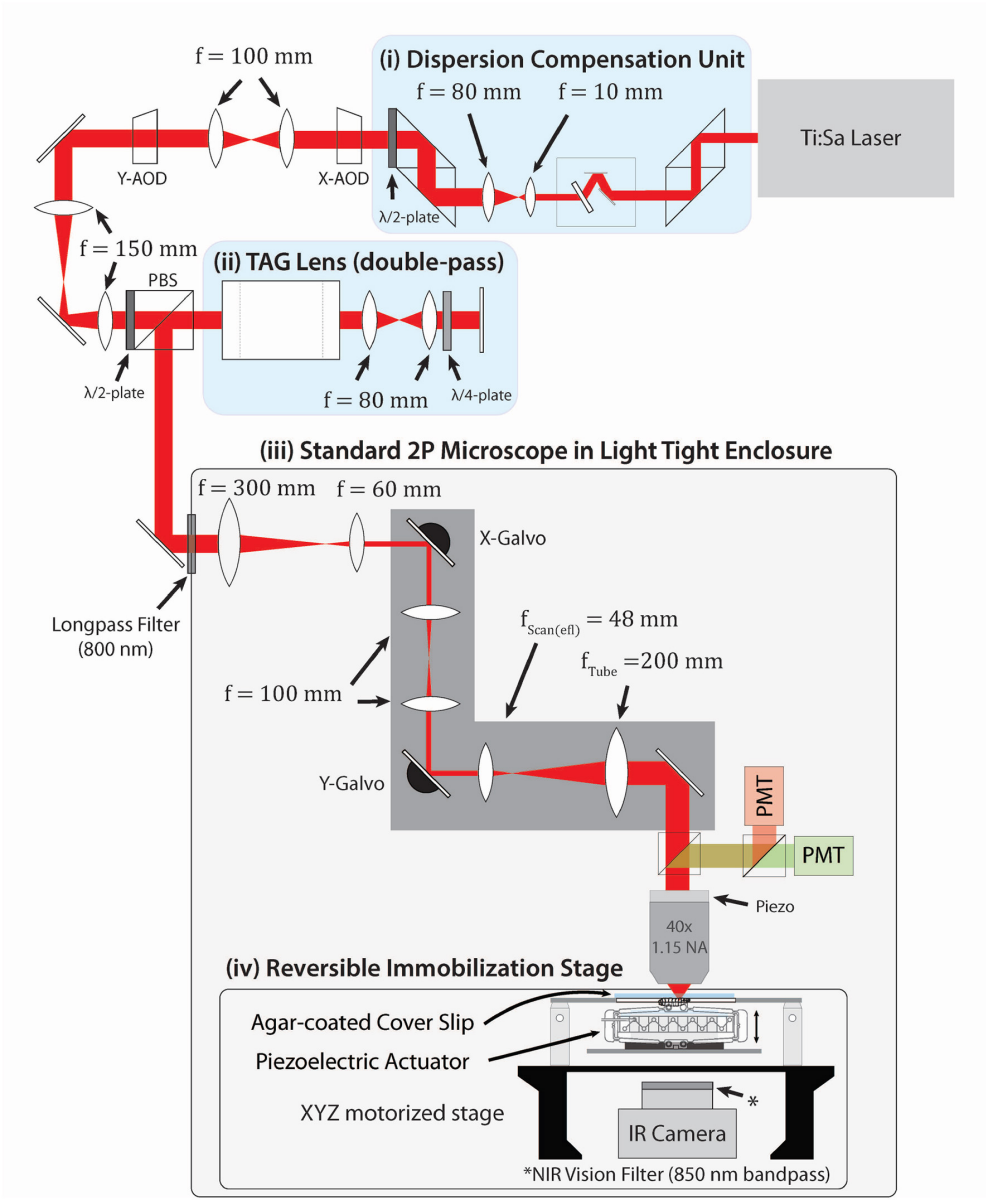
Optical layout of the system. Pulses with a central wavelength of 960 nm from a Ti:Sa laser first pass through a **(i)** dispersion compensation unit (DCU) (Yamaguchi et al., 2021) tuned to compensate for spatial and temporal dispersions introduced by later elements, then **(ii)** expanded to fill two AODs separated by a 4f relay for x and y deflection. The AODs are relayed onto a TAG lens in double-pass configuration **(iii)**. Elements **(i)–(iii)** form a combined random access x-y + resonant z 3D acousto-optic scanner. This scanner is conjugated onto a standard 2P microscope placed in a light-tight enclosure. The larva crawls on an agar-coated coverslip mounted to a 3-axis stage beneath the microscope (Reversible Immobilization Stage) **(iv)**. A piezoelectric “squisher” temporarily holds the animal in place prior to the start of tracking. Tracking software controlled by an FGPA updates neuron locations at 10 kHz; feedback to the stage every 25 ms keeps the neurons centered in the field of view. IR and 450 nm lasers are set above the stage to illuminate the larva and provide visual stimulus (not shown). A more detailed design of the stage is shown in Supplementary Figure S1.

Our microscope (Figure 1) combines two types of acousto-optic scanners. We position the focal spot in the X-Y plane using two orthogonally oriented AODs; for axial scanning, we use a TAG lens, which acts as an oscillator in optical power at ultrasonic (here 70 kHz) frequencies. Compared to two AODs alone, this scheme allows for rapid axial scanning. Compared to a four AOD lens, this setup is simpler to align and has higher optical throughput, at the cost of less flexibility in the achievable scan patterns. A Ti:saph laser tuned to 960 nm impinges first on a tunable dispersion compensation unit (Yamaguchi et al., 2021) combined with a beam expander that expands the beam to fill two 9 mm TeO_2_ AODs separated by a 4f relay. These AODs are relayed onto a TAG lens in a double-pass configuration. The beam is then shrunk 5x to fit on the galvos of a home-built two-photon microscope (Karagyozov et al., 2018). The system is constructed so that the AODs, TAG lens, and both galvos are all conjugate with the back aperture of the microscope objective.

The TAG lens control unit sustained a resonant oscillation at a user-specified amplitude and a frequency of approximately 70 kHz and output a synchronization signal used to determine the oscillation phase and hence the z-location of the focal spot. The x- and y- AODs were driven by amplified signals from a DDS evaluation board and controlled by custom FPGA software written in NI LabVIEW. The FPGA received input from the TAG lens driver and from the two PMTs, allowing the synchronization of x/y deflections and axial scan lines and real-time determination of the origin of each detected photon.

### 2.2. AOD transitions can be matched to TAG lens oscillations

During random access scanning with acousto-optic deflectors, the focal spot is moved discontinuously from point to point by changing the driving frequency of the piezo resonators. During the time it takes for the new wavefront to propagate across the crystal (the aperture of the deflector divided by the speed of sound, called the acoustic access time), two discontinuous waves exist in the crystal. As a result, the focal spot at the original location first lessens in power and disappears, then a new spot appears and grows in power at the new location. For other reported systems (Otsu et al., 2008; Grewe et al., 2010), the transition time—the time from loss of power in the first spot to gain of power in the second—is less than the full acoustic access time.

**Figure 2:**
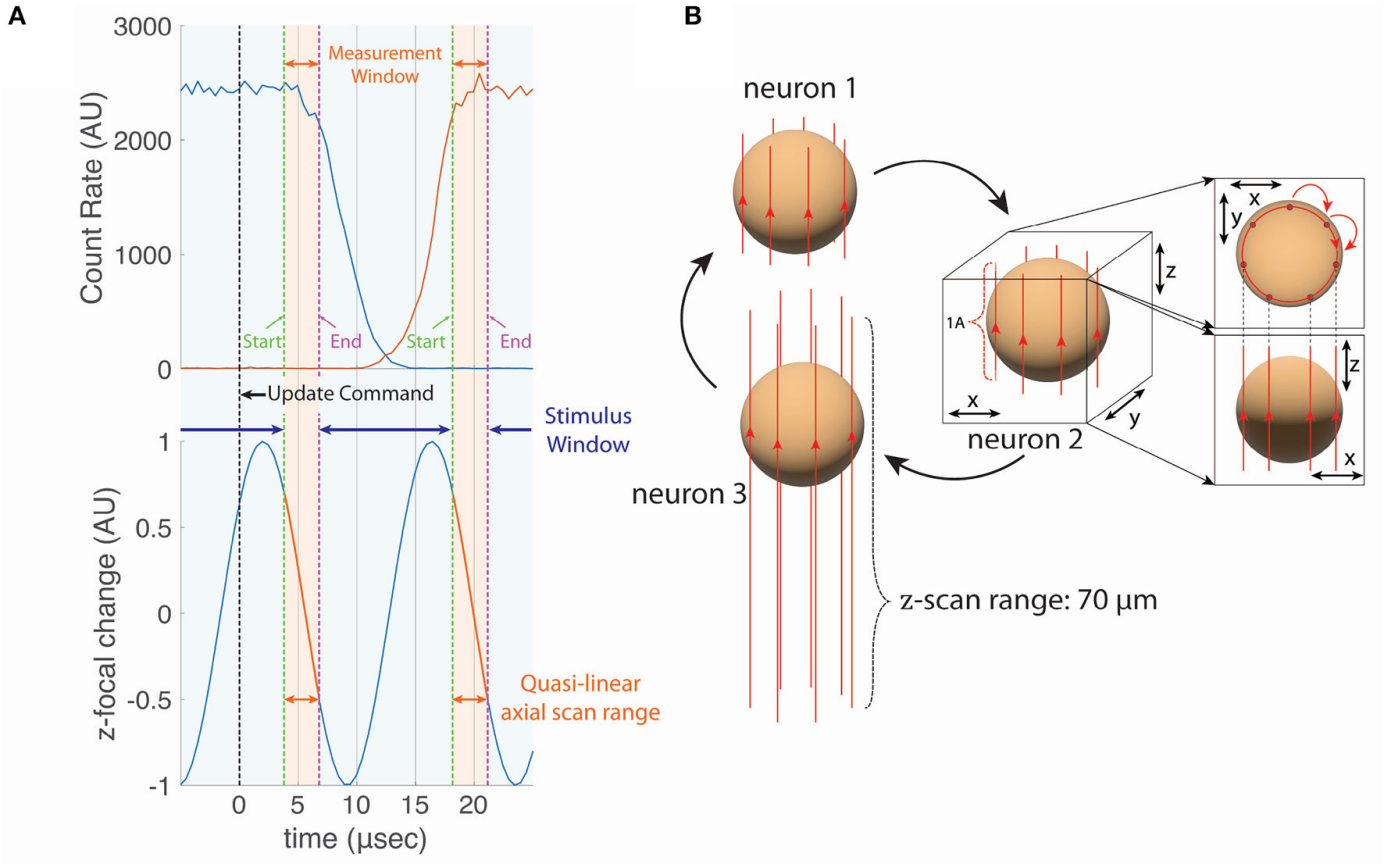
**(A)** The phase (position of the axial focal point) of the TAG lens with respect to the beam transition time of the AOD and the visual stimulation window. We collected the fluoresced photons during the quasi-linear axial scan range, which we defined to be a 50% of the TAG scan amplitude. The beam moves laterally during the Access Time. The visual stimulation was presented during the Stimulus Window to minimize the photon cross-talk. **(B)** The cylindrical scan for neuron tracking. Each vertical line around the sphere (i.e., cell body of the neuron) represents the trajectory of the axial focal point during one TAG cycle shown in **(A)**. The beam spot moves to the next line during Access Time. In this work, seven vertical lines were sampled per tracked neuron, resulting in a measurement rate of 10 kHz.

The acoustic access time for our deflectors was 13.5 μs. To measure the transition time, with the TAG lens turned off (no axial scanning) we set the AODs to alternately direct the laser focus onto a fluorescently labeled bead and into empty space 13.5 microns away. We recorded the rate of fluorescence emission vs. time from the transmission of the update signal (Figure 2A) for transitions off of the bead (blue line) and found that the fluorescence signal maintained ≥80% of its value for 7.5 μs following the signal. We also made the same measurement for transitions on to the bead (red line) and found that emission reached 80% of the steady state value 18 μs after the signal. Thus the transition time was ~10.5 μs.

When placed in a conjugate plane to the back of the microscope objective TAG lens creates a resonant axial scanner and shares the common disadvantage of all resonant scanners—the sinusoidal (in time) scan pattern concentrates the dwell time at the extremes of the scan—50% of the scan time is devoted to the outer 30% of the scan. In resonant scanning, it is common to either blank the excitation laser or discard a portion of the data at the extremes of the scan.

By happy coincidence, the particular resonant frequency of the TAG lens and the transition time of the AODs allowed us to minimize the combined effects of lost scan time due to discontinuous transitions and resonant scanning. Typically, we would use the central 70% of the TAG lens's spatial scan (50% of the temporal duration) for imaging and tracking (over this interval, the axial position of the focus is approximately linear in time, so we call it the linear scan range). Each full period can be represented as two half cycles, *trace* and *retrace*, in which the axial focus is moving in opposite directions. In the first half cycle, this linear scan range would represent 50% of the scanning time, or at the 70 kHz resonant frequency 3.57 μs; we would then discard the next 3.57 μs centered on the extreme of the axial range, before restarting imaging (in the reverse axial direction) in the second half cycle. Because the AOD transition time is 10.5 μs, we cannot accomplish the transition entirely at the extremes of the axial scan. But the time between the end of the linear scan range and the start of the linear scan in the next full cycle is 10.7 μs, almost exactly the same as the transition time. Thus, by appropriately synchronizing the AOD-update signals to the TAG lens phase (Figure 2B), it is possible to record an axial line in a new position on each trace half-cycle, at the cost of discarding the retrace signal.

This strategy achieves the maximum line sampling rate (70 kHz, the resonant frequency of the lens) with a “duty cycle” of 25%—that is 25% of the total experimental time is used for sampling (compared to 50% for a galvo-galvo-TAG lens microscope). An alternate approach (Supplementary Figure S2) would be to change the x-y sampling location on alternate TAG cycles. This would achieve a duty cycle of 25% at half the maximum line sampling rate (35 kHz) and would support AOD access times as long as 1.25 times the tag lens period (17.8 μs).

### 2.3. *In vivo* multineuronal recording in moving *Drosophila* larvae

To demonstrate the improved utility of the tracking microscope for recording neural activity in freely crawling larvae, we carried out three sets of experiments. We simultaneously recorded activity from multiple VNC interneurons, finding a relation between these neurons' activities and the instantaneous locomotion of the larva; we recorded from a central brain descending interneuron whose activity reflected the larva's behavioral state; and we recorded from bilateral visual interneurons whose activity was driven by stimulus presentation.

In all experiments, we labeled the target neurons with and recorded the fluorescence of a stable red indicator protein (hexameric mCherry; Shearin et al., 2014) and a green calcium indicator [GCaMP6f (Chen et al., 2013) or GCaMP7f (Dana et al., 2019)] or, for control experiments, a stable green indicator protein (hexameric GFP; Shearin et al., 2014). Movement of the neuron within the scan volume, deviation of the neuron from its estimated position, deformation of the brain, and scattering by the cuticle and intervening tissue, will all affect the recovered fluorescence. Both red and green fluorescence are excited by the same laser pulse and collected by the same objective, so these changes should all affect the recovered red and green fluorescence equally and not affect the ratio of green to red fluorescence, which is used as a measure of activity throughout the work.

#### 2.3.1. Simultaneous recording from three premotor neurons (A27h)

**Figure 3:**
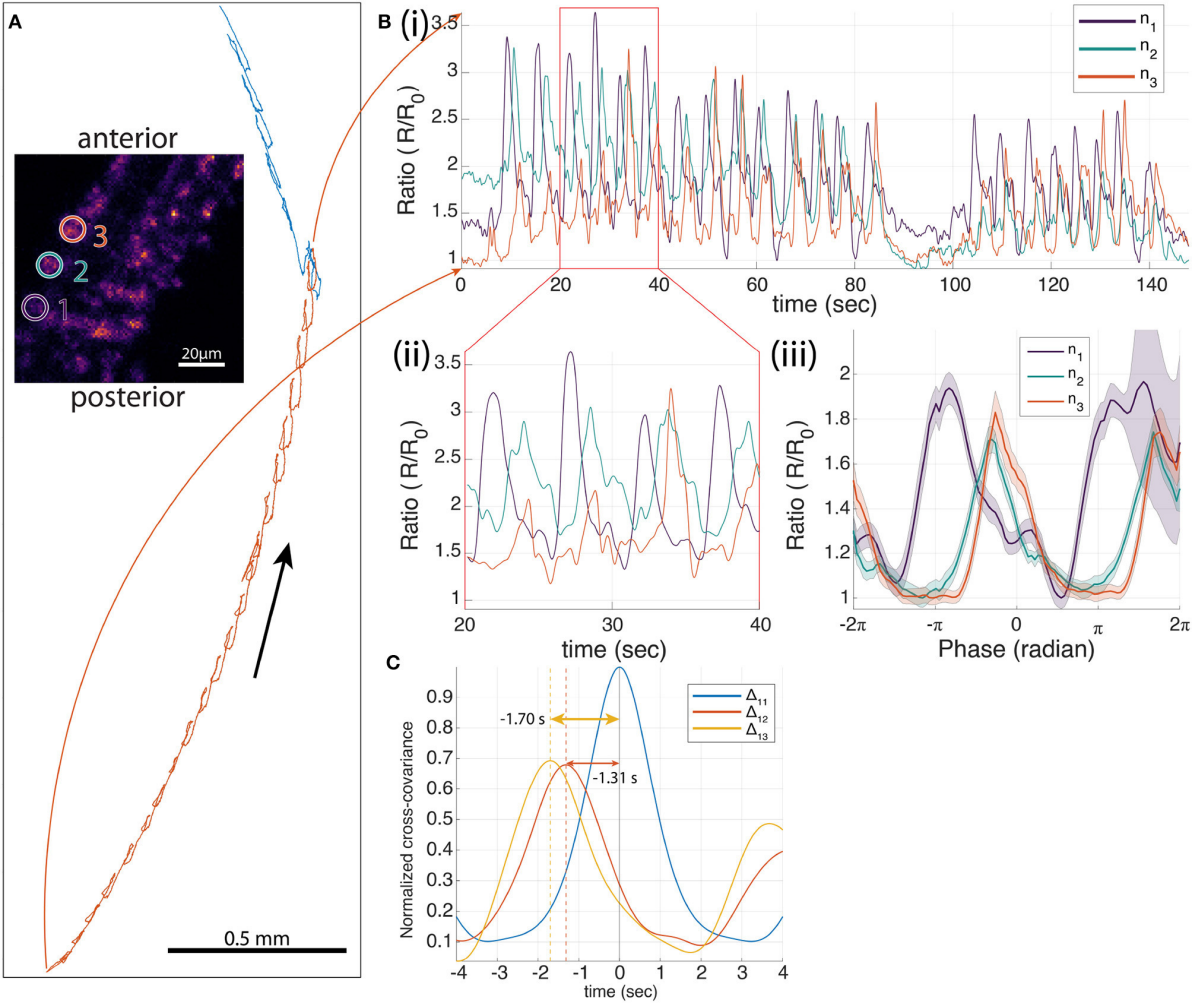
Three-neuron recording of A27 h>GCaMP6f;mCherry in a moving larva. **(A)** The trajectory of one neuron during forward crawling. In 214 s, 26 peristaltic cycles were observed. The inset image shows the 3D projection of the larval VNC. The three tracked neurons are indicated by white circles (1–3 from posterior to anterior) that correspond to *n*_1_ to *n*_3_ in [**(B)**(i–iii)]. **(B)** Ratiometric activity measure of each neuron. (i) One hundred and forty-eight second segment of the Ca^2+^ traces of neuron 1–3 (ratio of green to red fluorescence) during the selected time range that corresponds to the red highlighted portion of the trajectory in **(A)**. Twenty peristaltic cycles were observed in the 148 s. (ii) Ten second segment of the ratiometric activity of each neuron. (iii) Mean neural traces of 26 peristaltic cycles, where *t* = 0 is the time when the neuron was at the furthest back point in each peristaltic cycle. **(C)** Normalized cross-covariance between the ratiometric signals for the 26 cycles. The temporal difference between neurons 1–2, and 1–3 are 1.31 and 1.70 s, respectively.

Fushiki et al. (2016) showed that in a dissected preparation the premotor VNC interneuron A27h was activated synchronously with motor neurons in the same segment during fictive forward crawling (motor neuron activity propagating from posterior to anterior) but not fictive reverse crawling (motor neuron activity propagating from anterior to posterior). Using our previous tracking microscope, we confirmed that A27h is preferentially active in phase with the peristaltic cycle during forward but not reverse crawling, but we did not observe a progression of activity in A27h from anterior to posterior because we recorded from only single neurons (Karagyozov et al., 2018). Here, we recorded from three A27h neurons that are labeled with mCherry and GCaMP6f from three adjacent segments on the same side of the ventral nerve cord (VNC) while the animal was crawling (Figure 3A).

During forward crawling, the three tracked A27h neurons all showed periodic modulation of the ratio of green to red fluorescence, with activity in the most posterior neuron leading that of the middle neuron which in turn leads the most anterior neuron (Figure 3Bii). To test the relation of the activity to the peristaltic crawling, we aligned the ratiometric activity measure to the motion of the brain, setting the point in which the brain is farthest back to be *t* = 0 (Karagyozov et al., 2018). We found that the activity was synchronized to the peristaltic crawling cycle and progressed from posterior to anterior. Note that the alignment of the signals was done entirely using the center-of-mass motion of the tracked neurons; the fluorescence signals themselves were not used for temporal alignment.

**Figure 4:**
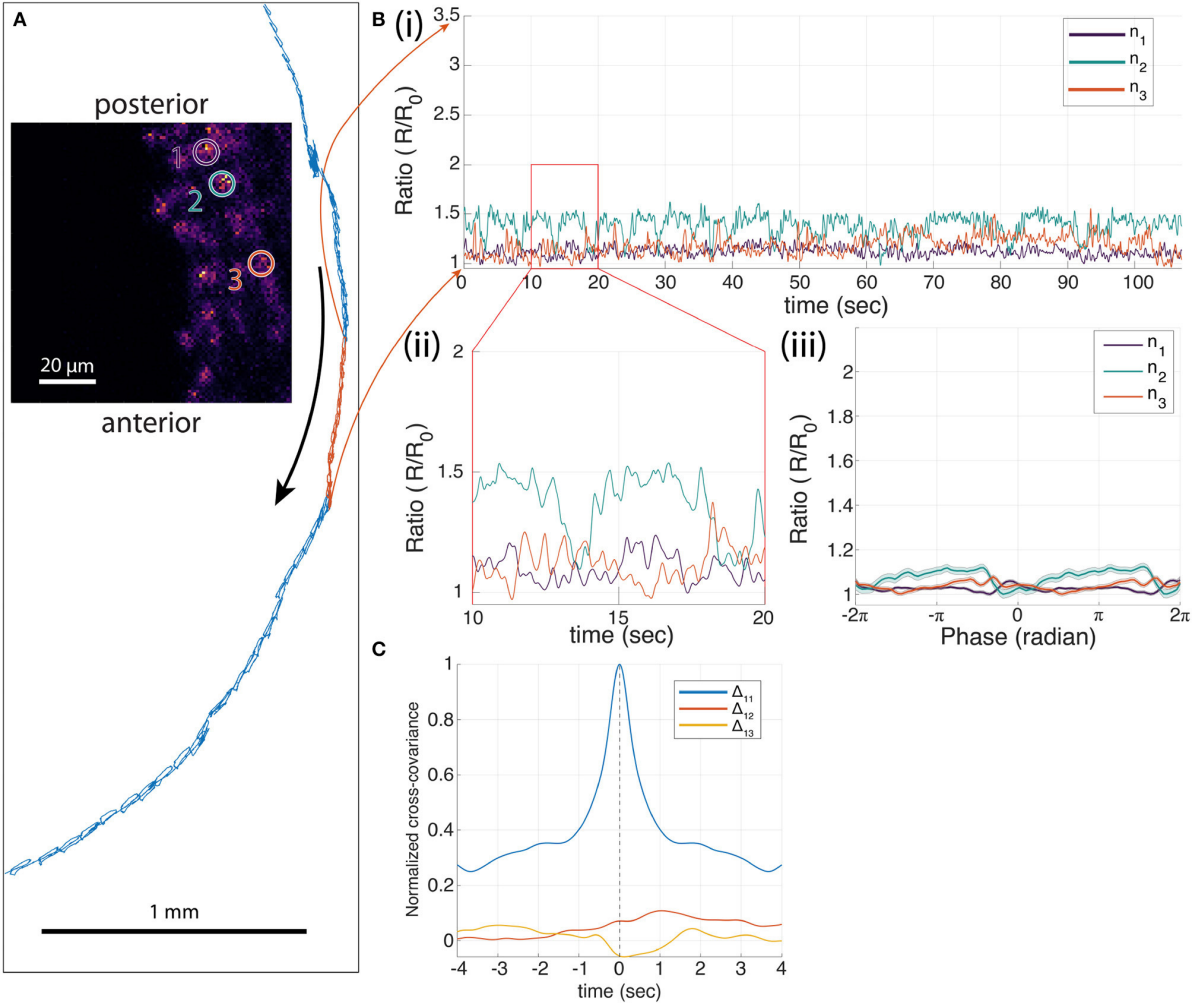
Three-neuron recording of A27h>GFP;mCherry in a moving larva. **(A)** The trajectory of one neuron during forward crawling in 586 s (blue trajectory) with 77 peristaltic cycles. The selected 107 s segment of the trajectory for the corresponding neural traces shown in [**(B)**(i)] is colored in red. **(B)** Ratiometric measures of each neuron. (i) One hundred and seven-second segment of ratiometric traces of neurons 1–3 (ratio of green to red fluorescence) with 18 peristaltic cycles, and (ii) 10-second segment of ratiometric measure of each neuron. (iii) Mean neural traces of the 77 cycles from the entire period shown in blue in **(A)**, where *t* = 0 is the time when the neuron was at the furthest back point in each peristaltic cycle. **(C)** The normalized cross-covariance between the neurons shows little temporal relationships.

We also measured the cross-covariance between the activity of the most posterior neuron and the other two neurons as well as the autocovariance of the neuron with itself. This analysis, which does not rely on the alignment of the signals to the peristaltic cycle, shows that the activity of the posterior neuron is highly correlated with the activities of the other two neurons and leads the other two neurons in anatomical order. In contrast, a separate experiment recording from three neurons labeled with mCherry and GFP (Figure 4), showed only small modulations of the green/red ratio with motion, and fluctuations in the ratio of one neuron were uncorrelated with fluctuations in the others.

We conducted similar experiments and analyses on A27h neurons labeled with GCaMP7f (Supplementary Figures S3–S7). These also showed a posterior-anterior activity progression aligned to the peristaltic crawling cycle, but the magnitude of the signals was smaller than for the GCaMP6f experiment (20–50% change in green/red ratio for GCaMP7f compared to 100–200% change for GCaMP6f). In comparison, GFP control experiments (Figure 4, Supplementary Figures S8, S9) show fluctuations in the green/red ratio on the order of 20%, but do not show the correlated posterior-anterior progression of the red/green ratio for each neuron visible in the GCaMP7f measurements.

#### 2.3.2. Moon crawler descending neuron

Optogenetic activation of the moon-crawler descending neurons (MDN) induces backwards crawling, and CaMPARI measurements show that MDN calcium levels are elevated during backward locomotion (Carreira-Rosario et al., 2018). The exact temporal relation between MDN activity and locomotion is unknown. For instance, we do not know whether changes in MDN activity lead or lag transitions between forward and backward crawling, and we do not know whether MDN is constitutively active during backward crawling, or if its activity is modulated with crawling.

**Figure 5:**
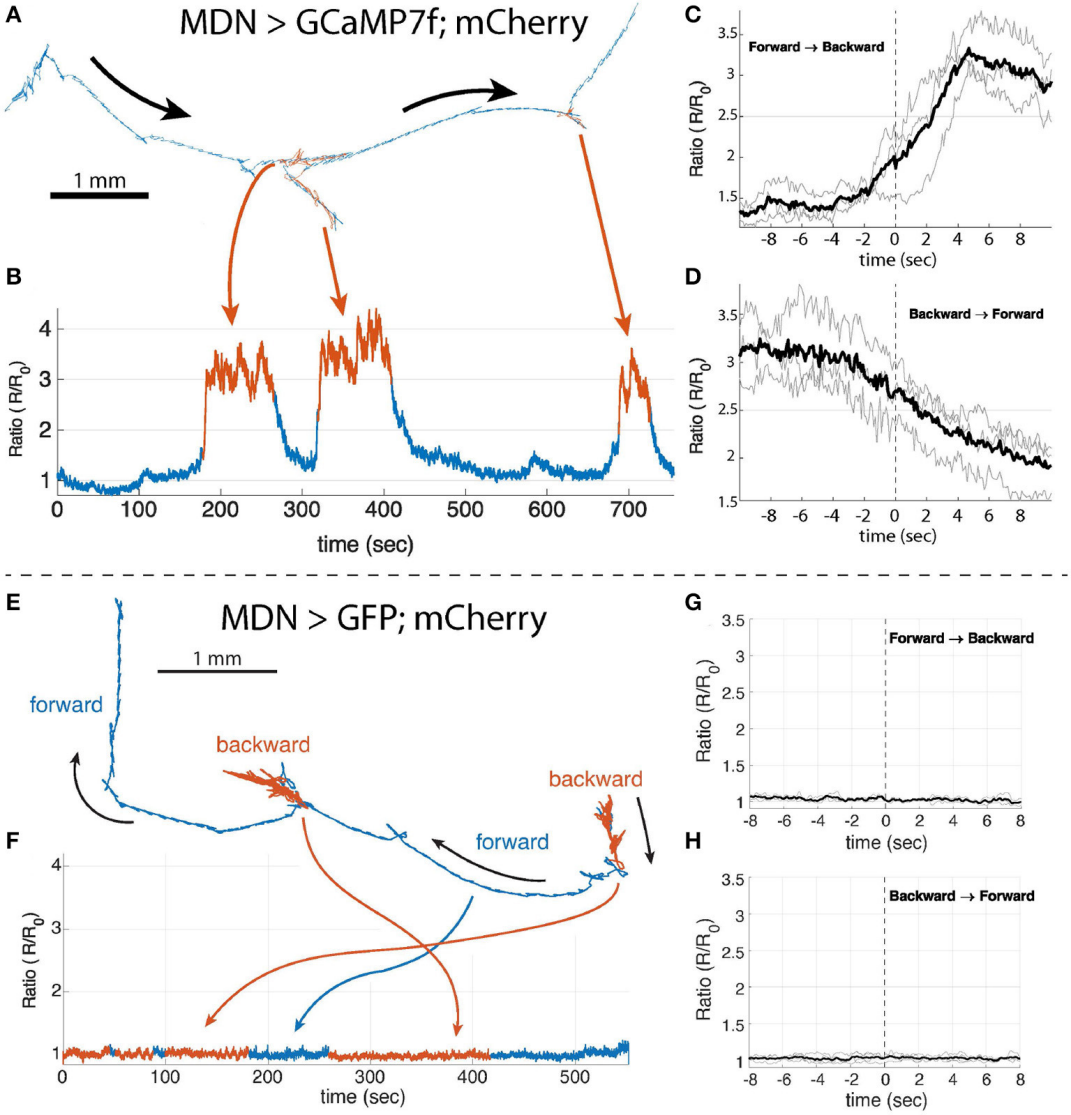
Single neuron recording of MDN>GCaMP7f;mCherry **(A–D)** and MDN>GFP;mCherry **(E–H)**. **(A, E)** The trajectory of the tracked MDN. Forward crawling period is shown in blue and the backward crawling period is shown in red. The blue and red indicate the period of forward and backward crawling, respectively. **(B)** The ratiometric Ca^2+^ activity measure of the MDN during the period shown in **(A)**. The Ca^2^+ concentration is high, the neuron is active, during backward crawling compared to forward crawling. **(C, D)** Show the mean Ca^2+^ trace (black lines) of the MDN when the larva changes the crawling direction from forward to backward or vice versa. The Ca^2+^ level of the MDN starts rising before the onset of the backward crawling and it starts decreasing before the onset of the backward to forward transition. *t* = 0 is the beginning of the first forward or backward peristaltic wave observed on the body wall from the behavior video that are manually selected from the behavior recording. **(F)** the ratiometric measure of GFP and mCherry in the MDN during the period shown in **(E)**. **(G, H)** the same analysis as in **(C, D)** with GFP.

We recorded from MDN in a larva crawling on an agar plate with the random access microscope. During the 6 min recording, we were able to observe multiple transitions of forward and backward crawling. Figure 5A shows the trajectory of the MDN; the corresponding Ca^2+^ activity measure (Figures 5B) shows increased activity during periods of reversal. Rising MDN activity predicted the transition from forward to backward crawling (Figure 5C), and falling MDN activity predicted the transition from backward (Figure 5D) to forward crawling. MDN activity was elevated without modulation during backward crawling. This pattern was repeated when four additional larvae were probed (Supplementary Figures S10–S13). In separate experiments, we saw no modulation in the ratio of green to red fluorescence of MDN neurons labeled with GFP and mCherry, during both forward and backward crawling (Figures 5E–H, Supplementary Figures S14–S16).

#### 2.3.3. Recording the response of bilateral visual interneurons in a moving larva

How larvae use temporal variations in light intensity to navigate away from light sources has been characterized extensively at the behavioral level (Sawin et al., 1994; Scantlebury et al., 2007; Sprecher et al., 2011; Keene and Sprecher, 2012; Kane et al., 2013; Gepner et al., 2015, 2018; Humberg et al., 2018); circuit mechanisms have been probed using genetic techniques (Busto et al., 1999; Hassan et al., 2000; Mazzoni et al., 2005; Keene et al., 2011; Humberg and Sprecher, 2018) and EM reconstruction (Larderet et al., 2017). However, understanding how activity in the visual circuit guides behavior is challenging. The larva's visual receptors are sensitive to even low levels of light (Kane et al., 2013) and located in close proximity to the central brain, so single-photon fluorescence techniques, including epifluorescence, confocal, and light sheet microscopy, with excitation wavelengths below ~650 nm (Salcedo et al., 1999) cannot be used for functional imaging in the visual circuit.

**Figure 6:**
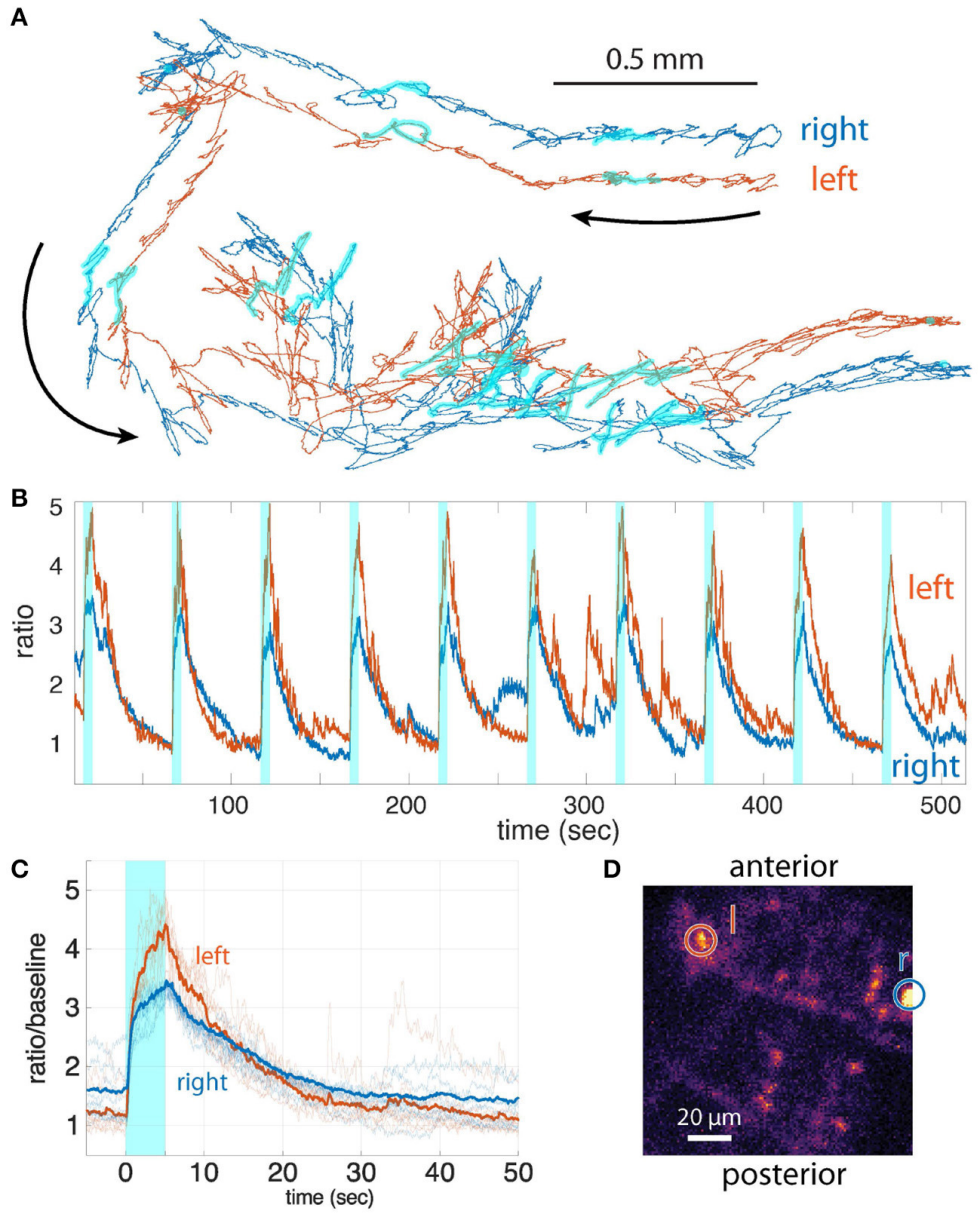
**(A)** The trajectories of the bilateral visual interneurons. The period of blue light illumination is highlighted in cyan. **(B)** Ratiometric activity measure vs. time for the two neurons for the period shown in **(A)**. Stimulus presentation is indicated by cyan shaded boxes. **(C)** Activities of the interneurons aligned to the onset of blue light (mean of *n* = 9 traces). **(D)** *z*-projection of the CNS; two tracked neurons are indicated by the circles (left and right, indicated by *l* and *r*, respectively).

We previously demonstrated that we could record the responses of visual interneurons to blue light presentation in behaving larvae. We modulated a blue laser so that light was presented only during the extremes of the TAG cycle; photons detected during this window were naturally discarded, eliminating cross-talk between the visual stimulus and the recorded fluorescence (Figure 6). For this microscope, we adopted the same strategy to avoid cross talk, taking advantage of a larger ‘dead' interval during the AOD transition (Figure 2 stimulus window).

Whether the larva decodes differences in the light responses of bilateral visual neurons to inform its navigational decision-making is currently unknown. To answer this question requires simultaneous recording from visual interneurons on both sides of the larva's brain. Our previous microscope could not record from such widely separated neurons. Using our new microscope, we recorded the light responses from bilateral visual interneurons separated by more than 100 μm in a freely crawling larva (Figure 6). We recorded stereotypical responses to light presentations that extended well beyond the offset of the stimulus and differed from left to right in both magnitude and temporal structure, all indications that the recorded responses represent neural activity and not cross-talk or motion artifacts. This demonstrates the feasibility of future experiments linking differences in bilateral sensory activity to behavioral outcomes.

## 3. Discussion

### 3.1. Advantages of random access two-photon microscopy for small moving animals

Numerous techniques using single-photon fluorescence have been described to record neural activity in untethered freely moving animals, including *C. elegans*, larval zebrafish, and hydra (Schrödel et al., 2013; Randel et al., 2014; Bouchard et al., 2015; Kotera et al., 2016; Nguyen et al., 2016; Venkatachalam et al., 2016; Cong et al., 2017; Dupre and Yuste, 2017; Kim et al., 2017). These methods have not translated to larval *Drosophila*. In the larva, spinning disk confocal microscopy was used to reveal transient bilateral differences in the aggregated activities of ~60 neurons driven by thermal activation of ectopically expressed TRPA-1 (Heckscher et al., 2015), and to record the temperature responses of peripheral thermosensory neurons in moving larvae during periods of “spontaneous quiescence” (Venkatachalam et al., 2016). SCAPE microscopy (Bouchard et al., 2015) was used to record activity from cuticular proprioceptors (Vaadia et al., 2019); recordings from the same neurons were also achieved using confocal and two-photon tracking microscopy (He et al., 2019). To date, recording from individual neurons in the CNS of a crawling larva has only been possible using two-photon tracking microscopy (Karagyozov et al., 2018).

Compared to other inertialess 2P microscopes, our microscope is optimized for 3D tracking. Methods that combine 2 AODs with mechanical refocusing (piezo translation or remote focusing; Botcherby et al., 2012) or electrically tunable lenses have bandwidths well under 1 kHz, limiting their utility in a real-time tracking system. Using only 2 AODs, it is possible to focus out-of-plane if the focal spot is rapidly scanned along particular lines (Vučinić and Sejnowski, 2007), or 3D point scanning can be achieved by a 2-AOD “SLM” if a laser with a repetition rate below the acoustic access time is used (Akemann et al., 2015, 2017, 2022). Full 3D random access scanning requires an acousto-optic lens (AOL) comprised of four AODs (Duemani Reddy et al., 2008; Kirkby et al., 2010; Katona et al., 2012; Nadella et al., 2016; Szalay et al., 2016).

For our system, for the two-AOD SLM, and for the four-AOD systems, the achievable sampling rate is limited by the acoustic access time of the AODs. Because of the resonant axial scan, we scan z-lines rather than individual points, providing information about the axial position of the neuron on every AOD update. Thus in principle, our microscope is capable of providing faster tracking than a random access point scanning microscope. Line scanning through the targeted neuron excites less fluorescence from the neuron than would random-access point scanning. However, in our continuous tracking application, bleaching of the tracked neuron rather than limited excitation power limits the achievable photon rate.

With two-photon random access microscopy, micron-scale motion can be compensated by scanning small local volumes rather than points (Katona et al., 2012; Fernández-Alfonso et al., 2014; Szalay et al., 2016; Akemann et al., 2022); larger movements require real-time tracking, which we previously achieved by combining galvanometric scanning in the x-y plane with resonant axial scanning (Karagyozov et al., 2018). More recently an all-acousto-optic tracker (Griffiths et al., 2020) was demonstrated using an AOL. In this more recent work, implanted reference beads were tracked rather than labeled neurons, but the method might be adapted to track cell bodies. While the AOL allows scan patterns our microscope cannot achieve, the simpler construction and alignment of our microscope may be appealing for applications focused on tracking.

### 3.2. Limitations on tracker performance

Our microscope was capable of tracking up to three widely separated neurons in a freely crawling larva. While this was an improvement over our previous microscope, we had hoped that removing inertial limits from the microscope would enable us to follow many more neurons.

#### 3.2.1. Single neuron with a diffusion prior

We begin by considering how the frequency at which a neuron's location is sampled affects the tracking uncertainty—i.e., do faster circles result in more accurate tracking? Due to shot noise, the uncertainty in a single measurement of the neuron's position is approximately $\sigma^{2}=R^{2}/N_{p}$, where *R* is the radius of the circular scan (in the axial direction, substitute *Z*, the axial extent of the neuron). The number of photons per measurement is Γ*t*_*neuron*_, where Γ is the rate of emission when the focal spot is over the neuron and *t*_*neuron*_ is the amount of time the neuron is sampled. Γ≲10^7^ Hz. If the amplitude of the axial oscillation due to the TAG lens were adjusted so that the quasi-linear regime (Figure 2A) exactly matched the neuron dimension, 25% of the total sampling time would be spent on the neuron. In fact, the amplitude of the axial oscillation must be much larger, to allow for axial movement of the neuron and imaging of neurons in multiple focal planes. If the linear regime encompasses about seven cell bodies (e.g., a peak-peak linear scan range of 50 microns and a cell body diameter of seven microns), then ~3.6% of the total scan time is spent within the neuron. If we assume that the total measurement time for a neuron is (as in this work) 100 μs, then $N_{p}≲10^{7}*3.6*10^{-6}=36$. If the radius of the circle is 3μm, then the measurement error is $\sigma =3/\sqrt{36}=0.5\mu$m. As is typical of a shot-noise limited process, this error scales inversely with the square root of measurement time.

Now consider a sequence of measurements of a diffusing object. To combine a series of measurements with error, we use a Kalman filter (Kalman, 1960; Karagyozov et al., 2018). Each measurement reduces the uncertainty in a neuron's estimated location, which then grows over time at a rate set by the diffusion constant *D*. After a long series of repeated measurements with the same error and timing, the uncertainties in the estimated location converge to constant values. Calling *P*^−^ the uncertainty (expressed as a variance) immediately before a measurement, *P*^+^ the uncertainty immediately after measurement, *Q* = *DΔt* the growth of uncertainty due to diffusion, and $M=R^{2}/N_{p}$ the measurement uncertainty, then


(1) $$\begin{array}{ll} P^{+} & =\frac{P^{-}M}{P^{-}+M} \end{array}$$

(2) $$\begin{array}{ll} P^{-} & =P^{+}+Q \end{array}$$

For a single neuron, the inter-neuron measurement interval Δ*t* is due to the time spent sampling the neuron. If γ = Γ*t*_*neuron*_/Δ*t*, the mean photon arrival rate over the sampling interval (accounting for the times when the focal spot is on and off the neuron), then $M=\frac{R^{2}}{\gamma \Delta t}$. Equations (1) and (2) can be solved to yield the maximum error as a function of sampling interval.


(3) $$\begin{array}{ll} P^{-} & =L^{2}\left(1+\frac{\Delta t}{\tau }\right) \end{array}$$

(4) $$\begin{array}{ll} L^{2} & =R\sqrt{\frac{D}{\gamma }} \end{array}$$

(5) $$\begin{array}{ll} \tau & =\frac{R}{2\sqrt{D\gamma }}=\frac{R^{2}}{2\gamma L^{2}} \end{array}$$

Equation (3) reveals the minimum uncertainty, *L*^2^ found in the limit of continuous sampling Δ*t* → 0, is a function of the scan size diffusion constant and average photon arrival rate. For typical values of *R* = 2.5μm, *D* = 100μm^2^/*s*, and γ = 3.6*10^5^,


(6) $$\begin{array}{lll} L^{2} & = & {\left(204\text{nm}\right)}^{2} \end{array}$$

(7) $$\begin{array}{lll} \tau & = & 208\mu \text{s} \end{array}$$

Once the sampling interval, Δ*t* gets below τ, there is a diminishing benefit to faster sampling. For instance, given the above parameters, the change from a sampling interval of 360 μs (Karagyozov et al., 2018) to 100 μs (this work) reduces the uncertainty from (337 nm)^2^ to (248 nm)^2^. Sampling at the minimum possible interval (3 points per neuron) of 43 μs, would reduce the uncertainty to (224nm)^2^.

The Kalman filter can be characterized by a Kalman gain. If *x*_*est*_(*n*) is the estimated position of the neuron following *n* measurements, and *x*_*m*_(*n*+1) is the location given by the (n+1) measurement, then


(8) $$\begin{array}{l} x_{est}\left(n+1\right)=\left(1-K_{g}\right)x_{est}\left(n\right)+K_{g}x_{m}\left(n+1\right);K_{g}=P^{-}/\left(P^{-}+M\right) \end{array}$$

In the limit of equally spaced measurements of equal uncertainty, *K*_*g*_ is constant, and *x*_*est*_(*n*) can be written as the result of an exponential filter on the measurements


(9) $$\begin{array}{l} x_{est}\left(n\right)=K_{g}\overset{n}{\underset{i=0}{\sum }}x_{m}\left(n-i\right)*{\left(1-K_{g}\right)}^{i} \end{array}$$

For our tracker, *K*_*g*_ is given by the sampling frequency Δ*t* and the characteristic time scale $\tau =R/(2\sqrt{D\gamma };$ Equation 15).


(10) $$\begin{array}{l} K_{g}=\frac{{\left(\Delta t/\tau \right)}^{2}+\Delta t/\tau }{{\left(\Delta t/\tau \right)}^{2}+\Delta t/\tau +2}\approx \frac{1}{2}\frac{\Delta t}{\tau },0\leq \frac{\Delta t}{\tau }\leq 1 \end{array}$$

We can use this expression to write the exponential filter (Equation 10) terms of time (*t*_*i*_≡*iΔt* and *t*≡*nΔt* is the time of the most recent measurement) as


(11) $$\begin{array}{l} x_{est}\left(t\right)\approx \frac{\Delta t}{2\tau }\overset{n}{\underset{i=0}{\sum }}e^{-\frac{t-t_{i}}{2\tau }}x_{m}\left(t^{\prime }\right) \end{array}$$

Thus a measurement made at one time continues to influence the estimate at future times for a period of approximately $2\tau =R/\sqrt{D\gamma }=1/\gamma *{\left(L/R\right)}^{2}$ that is independent of the sampling interval. In other words, once Δ*t* < τ, increasing the sampling rate does not decrease the latency of the tracker. Instead the latency is set by the rate at which photons are recovered from the tracked neuron (γ) and the desired accuracy of the tracker.

#### 3.2.2. Multiple neurons with a diffusion prior

Assume that *N* neurons are tracked independently, that each measurement takes a time Δ*t* and that the transition between neurons is instantaneous. For each tracked measurement of a tracked neuron the measurement error will be $M=\frac{R^{2}}{\gamma \Delta t}$ and the uncertainty will grow by *Q* = *D*(*NΔt*) between measurements of the same neuron. Substituting these values into the previous analysis will yield


(12) $$\begin{array}{lll} P^{-} & = & L^{2}\left(1+\frac{\Delta t}{\tau }\right) \end{array}$$

(13) $$\begin{array}{lll} L^{2} & = & R\sqrt{\frac{ND}{\gamma }} \end{array}$$

(14) $$\begin{array}{lll} \tau & = & \frac{R}{2\sqrt{ND\gamma }}=\frac{R^{2}}{2\gamma L^{2}} \end{array}$$

The Kalman gain calculation would remain the same, but because the measurement update rate would be reduced by a factor of 1/*N*, when written in terms of time, the exponential filter formulation (Equation 12) would yield


(15) $$\begin{array}{l} x_{est}\left(t\right)\approx \frac{\Delta t}{2N\tau }\overset{n}{\underset{i=0}{\sum }}e^{-\frac{t-t_{i}}{2N\tau }}x_{m}\left(t^{\prime }\right) \end{array}$$

In other words, to achieve the same positional uncertainty, the latency of the tracker must be increased by a factor of N. As long as $\Delta t<\frac{R^{2}}{2\gamma L^{2}}$, this result is approximately independent of sampling frequency.

The difficulty with multi-neuronal tracking using a diffusion prior arises due to this latency effect. In fact, in a crawling larva, temporally correlated movements characterized by velocity and acceleration dominate the motion, not diffusion. If the tracker does not update fast enough, the neuron may move so far between samplings that the next circular probe will not intersect the neuron and tracking will be lost. This occurs approximately when *Nvτ*>*R*, implying a maximum velocity


(16) $$\begin{array}{l} v_{max}\sim \frac{R}{N\tau }=\frac{2\gamma L^{2}}{NR} \end{array}$$

For reasonable choices of γ (the photon flux), *R* (the size of the tracked neuron), and *L* (the acceptable rms error in the estimate of each neuron's location), this estimate for *v*_*max*_ works out roughly to 1 cm/s for a single neuron. As the peak velocity of neurons in crawling larvae is typically on order of mm/s, this is consistent with our experience that the tracker is robust for single neurons but fails for large multiples.

This analysis does not include other effects that work against tracking. In particular, the irregular geometry of the neurons, inhomogeneous labeling, background auto-fluorescence, and off-target labeled structures near the target neuron all increase measurement error. The efficiency of fluorescence excitation and photon collection is lower far from the natural focal plane of the objective. When a single neuron is tracked, feedback to a piezo positioner on the objective maintains the neuron near the natural focal plane; when multiple neurons are tracked simultaneously, feedback places the center of mass at the focal plane, reducing the rate of photon emission (γ above) from each neuron.

#### 3.2.3. Modifications to the Kalman filter

To overcome limits on the velocity of a neuron in the diffusion only Kalman filter, it is natural to include both the position and velocity of the neuron as state variables, an approach adopted in our previous work (Karagyozov et al., 2018). Long distance motions (more than a few tens of microns) of the neurons result from translation of the whole brain and should therefore be highly correlated among the individual neurons.

Taken together, these suggest a model which tracks the position of each neuron relative to a center of mass together with the velocity of that center of mass. However, we found that a tracker based on such a model was less stable when tracking multiple neurons than the simple individual neuron tracker with a diffusive prior. We also failed to see an improvement using a purely diffusive tracker that included a center of mass term to introduce correlations between the neurons. Because we have only the tracker estimates of the positions and velocities of the neurons (i.e., we do not have an independent “ground truth” measurement of the motion), it is difficult to understand exactly why introducing a correlated velocity to the model did not improve performance; we suspect that a combination of anti-correlated motion (due to rotations), brain deformation, and beam deflection by the cuticle, none of which are properly modeled by any version of our Kalman filter, contribute to the lack of fidelity.

#### 3.2.4. Potential areas for improvement

In this work, we demonstrated that multi-neuronal tracking, even of widely spaced neurons, is feasible using an all acoustic deflection scheme. This opens up the possibility of implementing further improvements not possible with galvo-based scanning.

As the fidelity of tracking crucially depends on the photon detection rate, steps to increase this rate could improve tracking. For instance, the FPGA could be programmed to modulate the RF power to the AODs on a neuron-by-neuron basis to maintain the emission at the peak rate allowed by the PMTs, detection electronics, and photo-bleaching. Fluorescent beads with well understood geometry, brighter fluorescence and greater resistance to bleaching could be implanted in the brain and used to track center of mass motion or even to serve as a reference for registered volumetric imaging (Griffiths et al., 2020).

AODs permit complex sampling patterns not realizable with galvos, and it may be possible to design an improved sampling pattern (Fields and Cohen, 2011) that can accurately estimate the position of a neuron even if the initial estimate of the position is off by several microns; it might also be possible to adapt the sampling pattern on the fly based on the results of previous measurements. For example, neurons might be tracked using a model that generates a non-Gaussian probability distribution (Taghvaei et al., 2017); this distribution could be used to generate a maximally informative sampling pattern.

## 4. Conclusion

By combining two AODs with a TAG lens, we created an all-acousto-optic random access line scanning microscope. Using this microscope, we tracked individual neurons with a latency of 0.1 ms, a factor of 3.5 improvement on our previous microscope. In freely behaving animals, we recorded phasic activity from multiple VNC interneurons, behavioral-state encoding activity in a descending command neuron, and light evoked bilateral activity in visual interneurons. We confirmed a lack of motion artifacts in control experiments in which the same neurons were labeled with GFP showed no modulation during the same behaviors. More advanced methods will be required to overcome difficulties created by non-rigid deformation of the brain induced by crawling.

## 5. Methods

### 5.1. Microscope setup

We augmented our previously described microscope (Karagyozov et al., 2018) by combining two AODs (Gooch & Housego, Model: MD050-9S2V47-3-6.5DEG-WAA-X and MD050-9S2V47- 3-6.5DEG-WAA-Y), a custom-built tunable dispersion compensation unit (DCU) (Yamaguchi et al., 2021), and two galvanometric mirrors (Cambridge Technology Model 6210H) with the ultrasonic acousto-optic lens (TL25β.B.NIR, TAG Optics, Princeton, NJ; Karagyozov et al., 2018; Figure 1). The TAG lens (Kong et al., 2015) is used as a resonant axial scanner with the resonant frequency of 70 kHz with 11 mm aperture and 60 V maximum driving amplitude, which gives the axial range of approximately four diopters with 15 V (25%) driving amplitude. The excitation beam travels through the TAG lens twice by relaying the principal plane of the lens to itself by a mirror and a *f* = 80 mm relay (AC254-080-B-ML), which doubles the axial scan range from 35 to 70 μm (Figure 1ii).

The collimated 990 nm pulsed excitation laser with the 1/e^2^ diameter of Φ = 1.2 mm and the sech^2^ pulse width of 140 fs with 80 MHz repetition rate from Chameleon Ultra II (Tunable Modelocked Ti:Sapphire laser by Coherent) excites both GCaMP7f and mCherry (Drobizhev et al., 2009; Shearin et al., 2014; Dana et al., 2019).

The excitation laser first travels through the DCU and the beam is expanded by the 8 × beam expander (with *f* = 10 and 80 mm achromatic lenses: AC050-010-B-ML and AC254-080-B-ML) to Φ = 9.6 mm to compensate for the spatial dispersion and fill the aperture of the AODs. The beam is then polarized by an achromatic half-wave plate (AHWP05M-980) and relayed to the principal planes of the AODs (*x* and *y*) and TAG lens (*z*; Figure 1). The principle planes of the AODs and TAG lens are relayed to that of the objective (MRD77410 N40XLWD-NIR—40X Nikon CFI APO LWD NIR Objective, 1.15 NA, 0.59–0.61 mm WD). The objective is mounted on a piezo positioner (Nano-F 100S, Mad City Labs, Madison, WI) with 100 microns of travel. For experiments in this work, the power at the back aperture was typically 20 mW.

The objective is mounted on a Scientifica Multiphoton Detection Unit (2PIMS-PMT-25 B/G Raw), which contains a dichroic beamsplitter and short pass filter to direct fluoresced photons onto a second dichroic beamsplitter, separating them into red and green channels each detected by a separate PMT (Hamamatsu R9880U). The PMT outputs are digitized by Hamamatsu C9744 photon counting units.

Beam intensity control is achieved by modulating the RF power to the AODs. The system does not include a pockels cell. Other than the loss of power due to the disruption of the acoustic wavefront during transitions between spots, we do not blank the laser beam. During experiments we record the arrival time of every photon, as well as the state of the galvo mirrors, AODs, and synchronization signals from the tag lens. When recording volumetric images, this allows later reconstruction of the recorded volume at varying spatial and temporal resolutions (Har-Gil et al., 2018). In principle this full data set could be used to refine the tracker estimates of position and fluorescence, but in this work the tracker output, which estimates the position and reports a total number of photons every 100 μs, was used directly.

The optical elements after the TAG lens (galvo-mirrors, scan and tube lenses, objective, and PMTs) are enclosed in a custom-made light-tight enclosure, which was designed on AutoCAD 2017 with a plugin, AutoQuoterX^®^ II. The parts for the enclosure were purchased from 80/20 ^®^ Inc. The effective focal length of the scan lens is set to be 48 mm (with AC254-050 and AC254-100) to minimize the beam cropping.

To smoothly immobilize and release a larva in the beginning of the experiments to perform imaging and to select neurons, we designed an immobilization stage using two amplified piezo actuators with a travel range of 1150 μm ± 15% (THORLABS APF710 controlled by a K-Cube piezo controller, KPZ101) to control the level of compression (Figure 1iv, Supplementary Figure S1). This custom stage sits on an automated 3-axis motor-driven stage (MS-2000 XYZ, Applied Scientific Instrumentation). The XYZ stage receives FPGA command from a PID feedback loop running on a Windows PC [64-bit Windows 10 Pro with Intel(R) Core(TM) i7-8700K @ 3.70 GHz and 64 GB memory] to center the neuron to the focus of the objective at 40 Hz (every 25 ms).

Fibers coupled to an IR led (Thorlabs Fiber-Coupled LED, M850F2 with a 850 ± 8 nm bandpass filter, FB850-40) and a 450 nm laser (Thorlabs SM Fiber-Pigtailed Laser Diode LP450-SF15) with a 450 ± 2 nm bandpass filter (Thorlabs FB450-10) are positioned on the XYZ stage to illuminate the animal and provide visual stimulus, respectively. An IR camera (Basler acA640-90um with *f* = 60 and 150 mm achromats (AC254-060-B-ML and AC254-150-B-ML) and MV850/40—NIR Vision Filter) is set under the stage to record the behavior of the larva (Figure 1iv).

For tracking neurons, we used a Kalman filter (Kalman, 1960; Enderlein, 2000; Berglund and Mabuchi, 2005, 2006; Fields and Cohen, 2011, 2012) as previously described (Karagyozov et al., 2018) and extended to track multiple neurons independently. With the AODs, we can increase the tracking frequency up to 23 kHz with a minimal three-line scan per neuron. For all the experiments that we report here, the sampling frequency was set to be 10 kHz (seven scan points per neuron), an improvement to 100 μs latency from 360 μs using the previously developed galvo-based method (Karagyozov et al., 2018).

The microscope was controlled by custom software written in LabView. Except for the stage and the TAG lens inputs, microscope hardware (including galvos, AODs, objective piezo positioner, PMTs, and TAG lens synchronization signals) was addressed and read out by a multifunction i/o board (NI PXIe-7847) with an integrated FPGA (Xilinx Kintex-7 160T), also programmed with LabView. Real-time functions, including scan generation, tracking, image assembly, and stimulus delivery, were controlled by the FPGA.

### 5.2. Axial scanning with a tunable acoustic gradient lens

We use a tunable acoustic gradient (TAG) lens (Kong et al., 2015) as a resonant axial scanner in combination with the AODs to rapidly sample a volume around the cell bodies of the selected neurons. With the TAG lens and AODs, we create a cylindrical scan pattern about targeted neurons (Figure 2B) to track and record the activities from, which we implemented the previously developed method (Hou et al., 2017; Karagyozov et al., 2018).

The TAG lens is controlled by TAG Drv Kit 3.2 (TAG Optics, Princeton, NJ) and driven at a resonant driving frequency of ~70 kHz (69.34 kHz) with 25% driving amplitude (equivalent to 15 V). This provides a much faster axial scan compared to the motorized stage or mechanically vibrated objective with a piezo.

The axial scan range (Figure 2A TAG Power Δ*z*) is set by the driving amplitude. The range can be increased by increasing the power to the lens, limited by aberrations that arise when diverging/converging light impinges on the back aperture of the objective. The range can be decreased by turning down the driving power, limited by the inability of the driving kit/lens combination to maintain a resonant oscillation at low powers. Our optical train was designed to maximize the possible achievable scan range for a given driving power. If a lower scan range is desired, the lens can be used in single-pass configuration (lowering the range by 50%) or the magnification following the TAG lens can be adjusted (the axial scan post objective is proportional to 1/*M*^2^, where *M* is the spatial expansion of the laser beam between the TAG lens and the objective. Because the time spent imaging a volume or neuron of interest is inversely proportional the axial range, it is best to tune the range to map the anticipated axial extent of the structures of interest and the range of motion of tracked neurons. For all experiments in this work, the range was maintained at 70 μm.

Figure 2A shows the temporal relationship between the phase of the TAG lens and the AOD access time. The focus of the pulsed excitation laser moves around 70 μm in height (TAG Power Δ*z*), and we collect the emission from the two fluorescent proteins during a quasi-linear axial scan range of around 35 μm or for the 50% of the TAG amplitude for measurement. The blue visual stimulation light is provided outside of the measurement windows to limit the cross-talk of the photons.

### 5.3. Performance of AODs

The frequency bandwidth of the AODs is 30 MHz (frequency range: 35–65 MHz), and the acoustic velocity is ν = 617 m/s. For λ = 990 nm beam, the diffraction angle ranges from 56.16 to 104.3 mrad (Δθ_*d*_ = 48.1 mrad). Given the 40 × objective and the total magnification of 0.83 with the *f* = 300, 60, 48, and 200 mm relay pairs along the excitation path, the FOV is given by


$$\begin{array}{l} \text{FOV}=\Delta \theta_{\text{total}}\times f_{\text{obj}}\\ \;=48.1\text{mrad}\times 0.83\times 5\text{mm}\\ \;\approx 200\mu \text{m} \end{array}$$

However, the diffraction efficiency varies across the frequency range. we calibrated the RF amplitude to achieve uniform laser intensity across the FOV. The details of the flat-field correction are explained in Section 5.3.2. When we track or image the neurons, we use FOV of 50~100 μm.

#### 5.3.1. Detailed setup of the AODs

**Figure 7:**
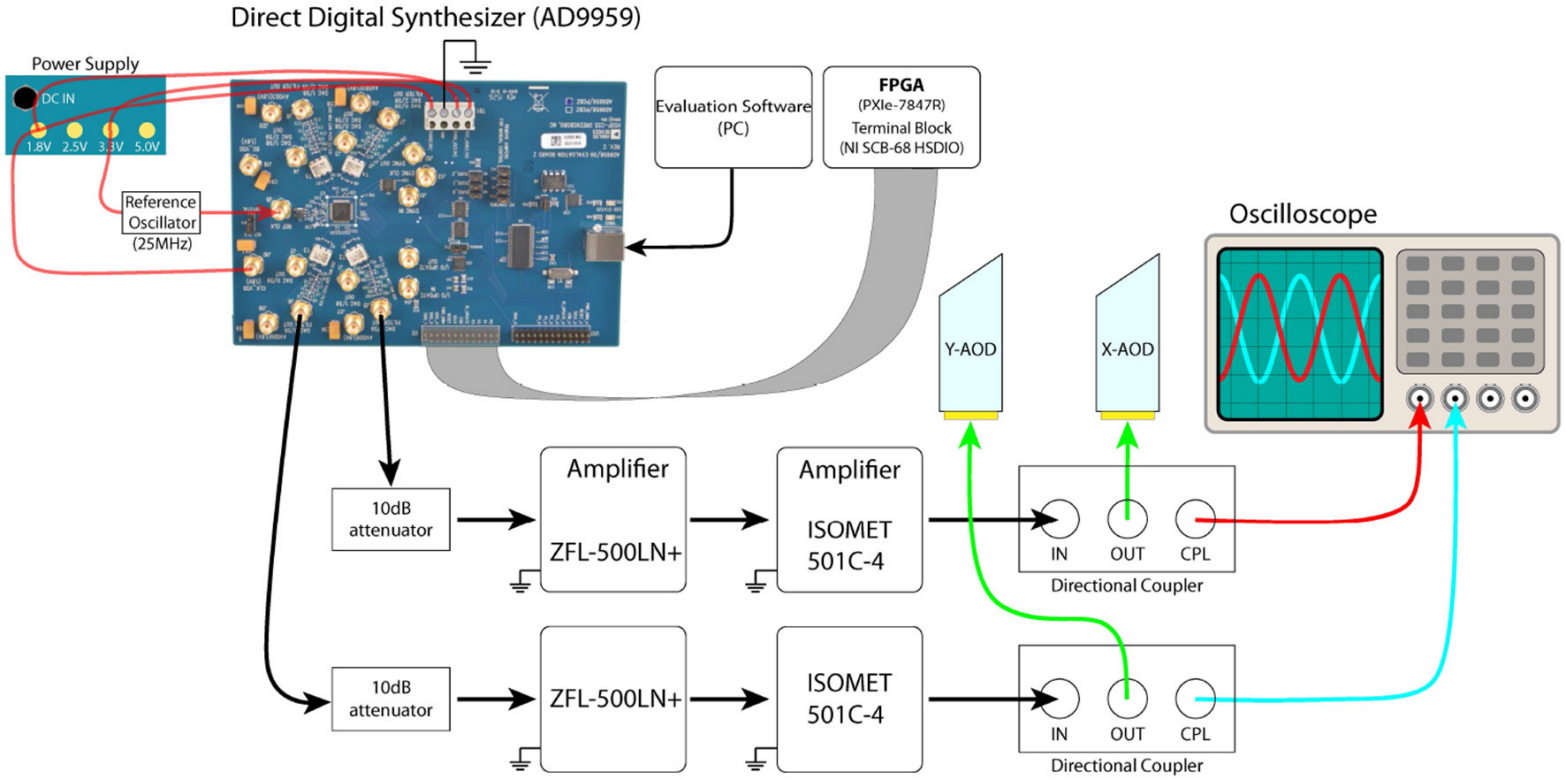
Electronics layout of the AODs. The direct digital synthesizer (AD9959) can be controlled by either the software (AD9959 evaluation software) via USB or by NI SCB-68 HSD through the serial port. The outputs from the directional coupler were connected to an oscilloscope (Tektronix TDS2024c) to monitor the frequency and amplitude of the RF signals in real-time.

The AODs are driven by a direct digital synthesizer (AD9959), which is powered by an ultra-low noise power supply (ABPSM-ULN-A) and uses a 25MHz pocket reference oscillator (Crystek CPRO33-25.000). This synthesizer is controlled by an FPGA (through National Instruments Shielded Connector Block, SCB-68 HSDIO and SCB-68A) and LabVIEW. The output RF signal from AD9959 is first attenuated by a 10dB attenuator (CATTEN-0100) and amplified by two RF amplifiers (ZFL-500LN+ and ISOMET Model 501C-4). The signal is then sent to a directional coupler (ZDC-20-3), which is introduced to reduce the reflection of the RF signal from the AODs (Figure 7). One output of the directional coupler is connected to an AOD and the other output is connected to an oscilloscope (Tektronix TDS2024c) to monitor the frequencies and amplitudes of the RF signals applied to the AODs in real time. The two amplifiers are powered with Longwei Electric DC Power Supply (LW-3010KDS).

#### 5.3.2. Flat field correction

**Figure 8:**
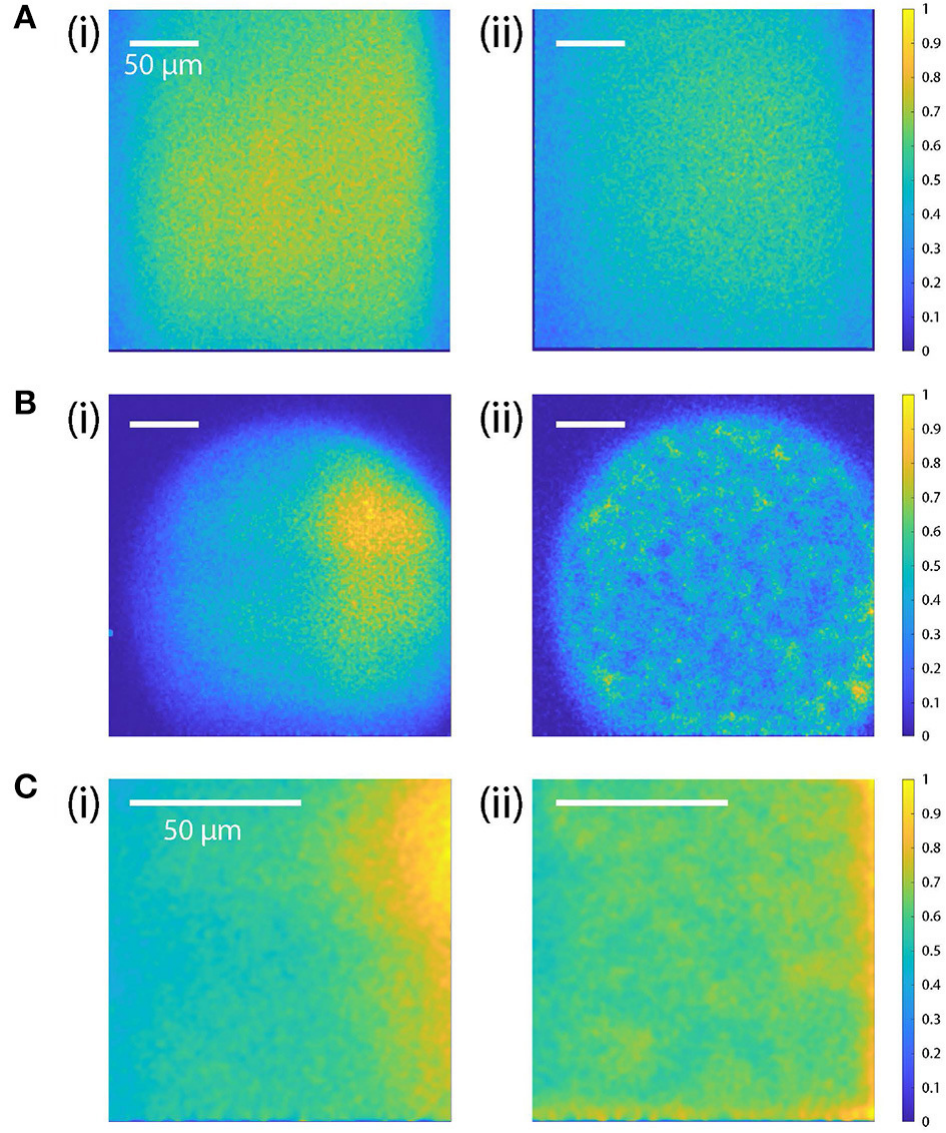
Comparison of the beam intensity profiles (flat-field correction). **(A)** Intensity profile with galvo-scanner with and without the TAG lens scanning (i and ii, respectively). The intensity profile across the FOV is relatively homogeneous, indicating that there is no change in beam intensity across the FOV. The overall beam intensity is higher when TAG lens is turned off. The RF frequencies applied to the two AODs are fixed at 50 MHz. **(B, C)** Beam profiles before (i) and after (ii) the field intensity correction with AOD-scan for different scan sizes: 250 × 250 μm and 100 × 250 μm for **(B, C)**, respectively. [**(B)**(i)]: For the maximum FOV (250 × 250 μm), the beam intensity drops significantly on the edge and has a circular inhomogeneous intensity profile. [**(B)**(ii)]: After the correction, the intensity profile is adjusted to have more homogeneous intensity over the circular area. [**(C)**(i)]: For a smaller FOV (100 × 100 μm), the beam intensity is higher on the right side, but it becomes more uniform after adjusting the amplitude of the RF signal [**(C)**(ii)]. The scale bars indicate 50 μm.

The beam intensity profiles are uniform when scanned with galvo-scanners (Figure 8A). In contrast, the diffraction efficiency of the AODs is not uniform across its scan range (35–65 MHz) resulting in inconsistent beam intensity and inhomogeneous photon count over the FOV (Figures 8B, Ci).

To correct this inhomogeneity, we calibrated the RF amplitude by measuring the light intensity of a fluorescent dye uniformly mixed in agar. The fluorescent sample was prepared with Φ = 0.1 μm dyed red aqueous fluorescent particles (CAT. NO. R100B, ThermoFisher Scientific) from Thermo Scientific (Cat#: R100B) and green fluorescein mixed in Apex Quick Dissolve LE Agarose (Cat#: 20-101QD). The red particles allowed the microscope to be focused within the gel, and the green gel served as a uniform reference. After recording the photon counts of the uniform fluorescent sample, we reduced the amplitude of the RF signal that correspond to the points with high photon counts (high laser intensity spots) to have more uniform intensity across FOV (Figures 8B, Cii). The required corrections to the driving amplitude were stored in a DRAM lookup table addressable by the FPGA.

### 5.4. Recording from crawling larvae

Larvae were placed on a coverslip coated with a thin layer of 4% agarose. The coverslip was inverted and attached to the stage under the microscope objective, so that the agar coated coverslip was between the ventral surface of the larva and the objective. To ease identification of the neuron(s) to be tracked and initialization of the tracker, the larva was briefly immoblized using gentle compression. Two amplified piezoelectric actuators (Thorlabs) pressed an acrylic disk against the dorsal surface of the larva while the larva was monitored using the infrared “behavioral” camera. This allowed micron-scale adjustment of the compression to provide the minimum needed to halt motion. For experiments recording from visual interneurons (Figure 6), the larva was placed with the dorsal surface against the coverslip.

Following immobilization, the larva's CNS was imaged using volumetric 2P microscopy. A pong or random access scan pattern was generated by the x-y AODs synchronized to the tag lens oscillation. The x and y galvos were used to adjust the center of the field of view. Two color 2D xy and xz projections were assembled on the FPGA and displayed in real time on the computer monitor. The arrival time of every photon along with the pointing direction of the galvos and AODs and the start time of every tag oscillation were written to disk for use in later image assembly.

Once the neuron(s) to be tracked were located in the field of view, the microscope operator would click on each neuron to be tracked in the control software, identifying the location for initialization of the tracker. A low-speed version of the tracking algorithm was used to correct for small motions of the neurons during this process. Once the operator was satisfied with the marked neuron locations, the tracker was engaged and the microscope began high-speed tracking with feedback, described in the next section.

After the tracker “locked-on” to the neurons, the compression was released slowly by adjusting the voltage applied to the piezeo actuators until the larva began crawling freely.

### 5.5. Tracking

The tracking algorithm described in Karagyozov et al. (2018) was used with minor modifications. We define the z-axis of our coordinate system to be parallel the axis of the objective, meaning the x and y axes are parallel to the focal plane of the objective. To determine each neuron's location, the AODs directed the focal spot in a circle of radius *R*, typically 3μm in x and y around the putative center of the neuron. In Karagyozov et al. (2018), the x-y scan was generated using galvos which created a continuous path in plane and limited the frequency of the circle to <3,000 rev/s. In this work, AODs positioned the focal spot at a discrete number of x-y locations around the circle, updating once per TAG cycle (sampling rate 70,000 spots/s); we chose 7 points per circle, resulting in 10,000 rev/s. Coincident with the x-y scan, the TAG lens created a resonant z (axial) oscillation of the focal spot with a peak-peak amplitude of ~70 μm.

The arrival time of each photon was recorded during the circle, correlated with the position of the AOD and tag lens and used to estimate the neuron's location. The TAG scan range extended ±35μm from the natural focal plane; only photons emitted from within ±*Z* (typically 5μm) of the estimated z-location of the neuron were used to estimate the neuron's location. Because the neuron was labeled quasi-uniformly with both red and green fluorescent protein, both red and green photons were used. Following each circle, the location of the new neuron was updated; full details of the calculation are in Karagyozov et al. (2018). Here we reprise the main themes.

With only a single scan, the best estimate of the neuron's location is the center of mass of the emitted photons ${\vec{x}}_{est}=\frac{1}{N_{p}}\sum \left(x_{i},y_{i},z_{i}\right)$, where *N*_*p*_ is the number of detected photons and (*x*_*i*_, *y*_*i*_, *z*_*i*_) is the point of origin of the *i*^*th*^ photon. Assuming *R, Z* were chosen appropriately to match the size of the neuron, the error of this estimate, due to shot noise, is approximated by $\sigma_{x}=\sigma_{y}=R/\sqrt{N}$ and $\sigma_{z}=Z/\sqrt{N}$ (Karagyozov et al., 2018).

To combine sequential uncertain measurements, we used a Kalman filter Kalman (1960); Karagyozov et al. (2018). Assume that all errors are Gaussian and following *i* measurements, the neuron's x-location and uncertainty are given by *x*_*i*_±σ*i*. A time Δ*t* later, the neuron's location is measured again. After Δ*t* but before applying the measurement, the best estimate of the neuron's x-location is unchanged *x*_*i*+1|*i*_ = *x*_*i*_ but the uncertainty has increased $\sigma_{i+1|i}^{2}=\sigma_{i}^{2}+D\Delta t$. Note that although *D* has units of a diffusion constant, because the motion is non-diffusive, *D* is best understood as a parameter that adjusts the responsiveness of the tracking system. Assume the new measurement places the neuron at *x*_*m*_±σ_*m*_. We combine the measurement with the previous estimate, weighting them inversely according to their errors. *x*_*i*_ = (1−*K*)*x*_*i*+1|*i*_+*Kx*_*m*_, $K=\frac{\sigma_{i+1|i}^{2}}{\sigma_{i+1|i}^{2}+\sigma_{m}^{2}}$. The combined error is now $\sigma_{i}^{2}=\left(1-K\right)\sigma_{i|i+1}^{2}=\frac{\sigma_{i|i+1}^{2}\sigma_{m}^{2}}{\sigma_{i|i+1}^{2}+\sigma_{m}^{2}}$. This new estimated location is used as the basis for the next round of measurements.

In this and prior (Karagyozov et al., 2018) work, we tracked motion along each axis separately (i.e., the estimate of the y-location did not include any information about the x-location of the neuron or of the emitted photons). While extension to tracking both position and velocity is straightforward (Karagyozov et al., 2018), in this work we tracked position alone.

#### 5.5.1. Feedback

Following each measurement, the estimated position of the measured neuron was updated. We used this updated location to calculate the location of the next measurement scan. Other layers of feedback served to keep the tracked neuron(s) centered and in the most effective range of the acousto-optic elements. From fastest to slowest: the x-y galvo deflectors were directed to the estimated mean location (estimated center of mass) of the tracked neurons, the piezo positioner on the objective was set to bring the estimated z- center of mass to the natural focus of the objective, and the stage was moved in all three axes to bring the tracked center of mass to the natural central position (galvos centered and objective piezo at half range) of the microscope.

#### 5.5.2. Quantification of fluorescent signals

The tracker records the number of red and green photons recorded from each tracked neuron with each tracking cycle. For each neuron, we define two parameters λ_*red*_(*t*) and *R*(*t*), the ratio of green to red fluorescence. The log probability of observing a particular sequence of green *n*_*g*_(*t*) and red *n*_*r*_(*t*) photon counts is given by Poisson statistics


(17) $$\begin{array}{l} \;\log(P)=\sum_{i}\log(\lambda_{r}(t_{i}))*n_{r}(t_{i})-\lambda_{r}(t_{i})\Delta t\\ +\log(R(t_{i})\lambda_{r}(t_{i}))*n_{g}(t_{i})-R(t_{i})\lambda_{r}(t_{i})\Delta t+C_{i} \end{array}$$

where Δ*t* is the sampling time and *C*_*i*_ = log(Δ*t*)(*n*_*r*_+*n*_*g*_)−log(*n*_*g*_!)−log(*n*_*r*_!) contains constants that do not depend on λ_*r*_, *R*

We apply a prior probability that the logarithm of intensity changes diffusively:


(18) $$\begin{array}{l} \log(P_{prior})=\sum_{i}-\frac{{\left(\log(\lambda_{r}(t_{i+1}))-\log(\lambda_{r}(t_{i}))\right)}^{2}}{4D_{1}(N\Delta t)}\\ \;-\frac{{\left(\log(R(t_{i+1}))-\log(R(t_{i}))\right)}^{2}}{4D_{2}(N\Delta t)}+C_{2} \end{array}$$

where *N* is the number of neurons tracked, *D*_1_ = 0.1, *D*_2_ = 0.0001 are parameters that determine the smoothness of the resulting fits, and *C*_2_ contains normalization constants that do not include λ_*r*_ or *R*. We assign *D*_2_<*D*_1_ based on our prior belief that fluctuations due to factors other than calcium dynamics should be faster than the variation due to calcium dynamics. This model is equivalent to the Stochastic Point Process Smoother (Eden et al., 2004) used in our previous work (Karagyozov et al., 2018). We minimize −log(*P*)−log(*P*_*prior*_) using the matlab function “fminunc”; the fit value of *R*(*t*) yields the ratiometric activity measure, while λ_*r*_(*t*) and *R*(*t*)λ_*r*_(*t*) yield the red and green rate estimates, respectively.

#### 5.5.3. Ratiometric baseline correction

The red and green indicators bleached at different rates, causing a long duration shift in the ratiometric intensity baseline. To correct for this, we found the ratiometric baseline by fitting the ratiometric measure to an exponential function [*r*_*base*_ = *a*exp(*bt*)] using a truncated cost function that discards large upward deviations. The baseline corrected ratiometric measure shown in all figures (ratio/baseline) is the instantaneous estimate of the ratio divided by this baseline.

#### 5.5.4. Cross-covariance

The normalized cross-covariance (shown in Figures 3, 4, Supplementary Figures S3–S9) is calculated as


(19) $$\begin{array}{l} \Delta_{ij}\left(\tau \right)=\frac{\int dt\Delta r_{i}\left(t\right)\Delta r_{j}\left(t-\tau \right)}{\sqrt{\int dt\Delta r_{i}{\left(t\right)}^{2}\int dt\Delta r_{j}{\left(t\right)}^{2}}} \end{array}$$

where Δ*r*_*i*_(*t*) and Δ*r*_*j*_(*t*) represent the deviation from the mean ratio for the *i*^*th*^ and *j*^*th*^ neurons, respectively.

### 5.6. Evaluation of optical performance

#### 5.6.1. PSF quality/point spread function measurements

Dyed red aqueous fluorescent particles with a diameter of 0.1 μm (CAT. NO. R100B, ThermoFisher Scientific) were used to measure the point spread function (PSF) of the system. Beads were embedded in a 1% agarose solution and the agarose was mounted on a slide with a coverslip placed over it.

**Figure 9:**
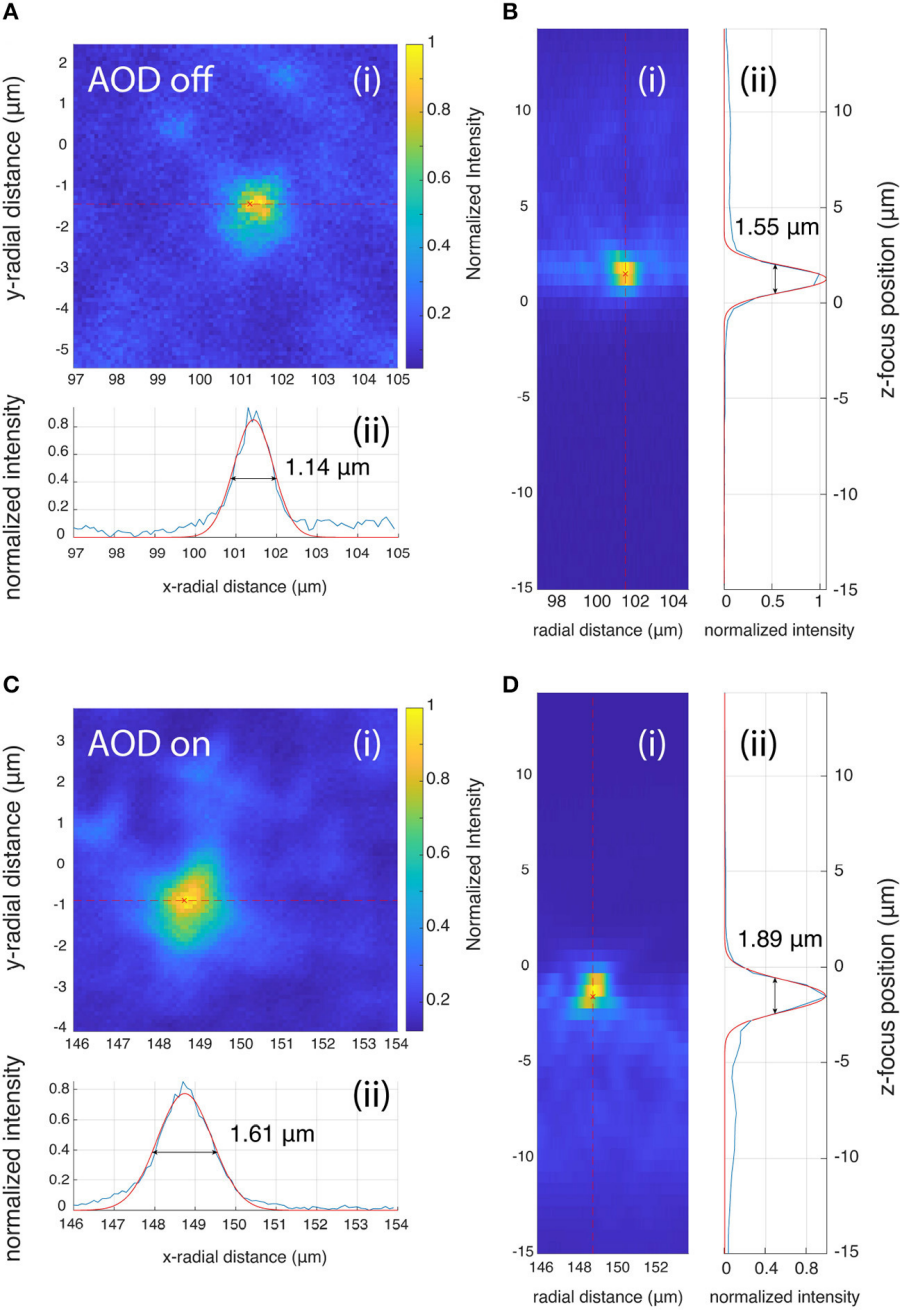
PSF measurements when AOD is off **(A, B)** and on **(C, D). (A, C)** Lateral PSF, **(B, D)** Axial PSF analysis. Both the lateral and axial FWHM are larger when the AODs are used for scanning (1.61 and 1.89 μm for the lateral and axial PSF) compared to when the RF frequency of the AOD is fixed at 50 MHz and the galvo mirrors are used for scanning (1.14 and 1.55 μm for the lateral and axial PSF).

The image stacks of the beads were acquired at 0.1 × 0.1 × 0.6 μm^3^ voxel resolution. Each bead stack was resliced into XY and YZ stacks and a maximum intensity projection was performed (Figure 9). The maximum intensity projections of image stack on the XY and YZ planes were then used to perform the FWHM measurements. A line was drawn through the center of the bead in the lateral and axial directions (Figures 9A–D). Pixel intensities along that line were fit with a Gaussian, and the FWHM values were determined from the width of the Gaussian fits.

Both the lateral and axial PSFs increased when the beam was scanned with the AODs, but the change is <0.5 μm, which does not affect the tracking accuracy.

#### 5.6.2. Random access volumetric imaging

To test whether we fully understood the timing of our microscope, we scanned a fixed sample volume using a pseudo-random *x*-*y* scan pattern, achieved by random permutation of a raster scan pattern. If we did not understand the location of the focal spots at all times, then the reconstructed image would be scrambled, and if we did not properly time the transitions to the TAG phase, the beam would fail to focus on the region of the sample we were imaging and we would see a dim or non-existent image. In fact, the volumetric image created by random-access AOD scanning matched that created by galvo-galvo raster scanning. Random access volumetric scanning enhances functional imaging experiments by removing correlations between a neuron's location in the volume and the time at which it is sampled.

### 5.7. Fly husbandry

The following strains were used: A27h-GAL4 (R36G02-GAL4, Bloomington 49939, Fushiki et al., 2016), MDN-GAL4 (SS01613-GAL4, gift of Albert Cardona, Carreira-Rosario et al., 2018), Tim-GAL4 (gift of Simon Sprecher), UAS-6xmCherry (Bloomington 52268, Shearin et al., 2014), UAS-6xGFP (Bloomington 52261, Shearin et al., 2014), UAS-GCaMP7f (Bloomington 80906, Dana et al., 2019), UAS-GCaMP6f (Bloomington 42747, Chen et al., 2013).

Males of the driver line were crossed against virgin females containing both fluorescent reporters (UAS-6xmCherry on 3, UAS-indicated green indicator on 2) and allowed to lay eggs on 60 mm egg collection plates. Larvae were used as second instars (verified by size and spiracle morphology) 48–72 h AEL.

## Data availability statement

The raw data supporting the conclusions of this article will be made available by the authors, without undue reservation.

## Author contributions

AY: design, construction, testing, experiments, data analysis, and wrote the manuscript. RW: software. PM: design, construction, and experiments. DK: construction. MM: design. MG: design, construction, data analysis, and wrote the manuscript. All authors contributed to the article and approved the submitted version.
