# Supplementary material for: Multi-neuronal recording in unrestrained animals with all acousto-optic random-access line-scanning two-photon microscopy
Source: Front Neurosci. 2023 Jun 14;17:1135457. doi: 10.3389/fnins.2023.1135457 (PMC10303936; doi:10.3389/fnins.2023.1135457)
Supplement: Supplementary file 1 [file Data_Sheet_1.pdf]

Supplementary Material

Multi-neuronal Recording in Unrestrained Animals with All Acousto-Optic Random-Access Line-scanning Two-photon Microscopy

Akihiro Yamaguchi, Rui Wu, Paul McNulty, Doycho Karagyozov, Mirna Mihovilovic Skanata, and Marc Gershow*

***Correspondence:** Corresponding Author: marc.gershow@nyu.edu

1 SUPPLEMENTARY FIGURES AND TABLES

1.1 Supplementary Figures

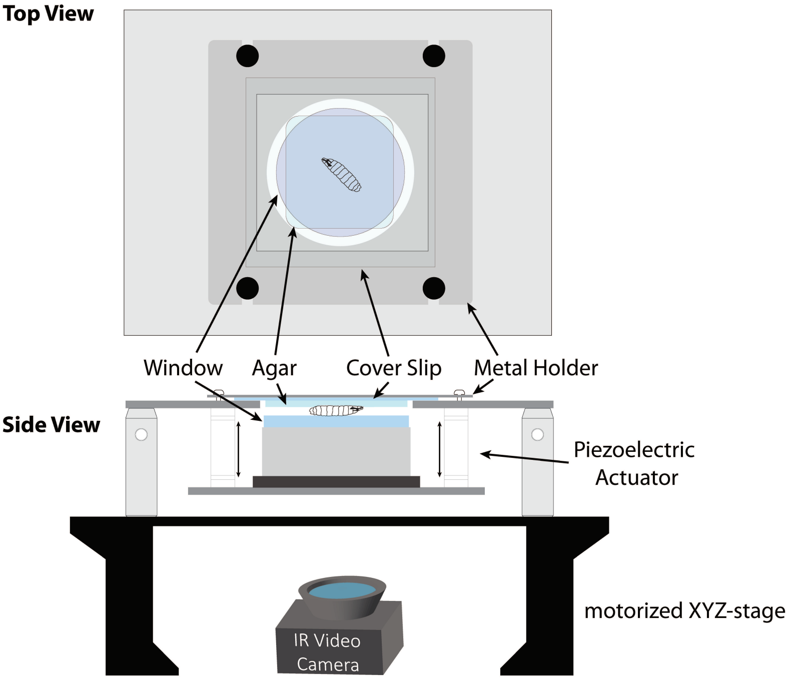

Figure S1. Immobilization stage design. The larva crawls on the agar place with their ventral side up. In a few experiments, the larva is set to crawl on the window with their ventral side down. We kept the agar wet and added water around the larva to match the refractive index.

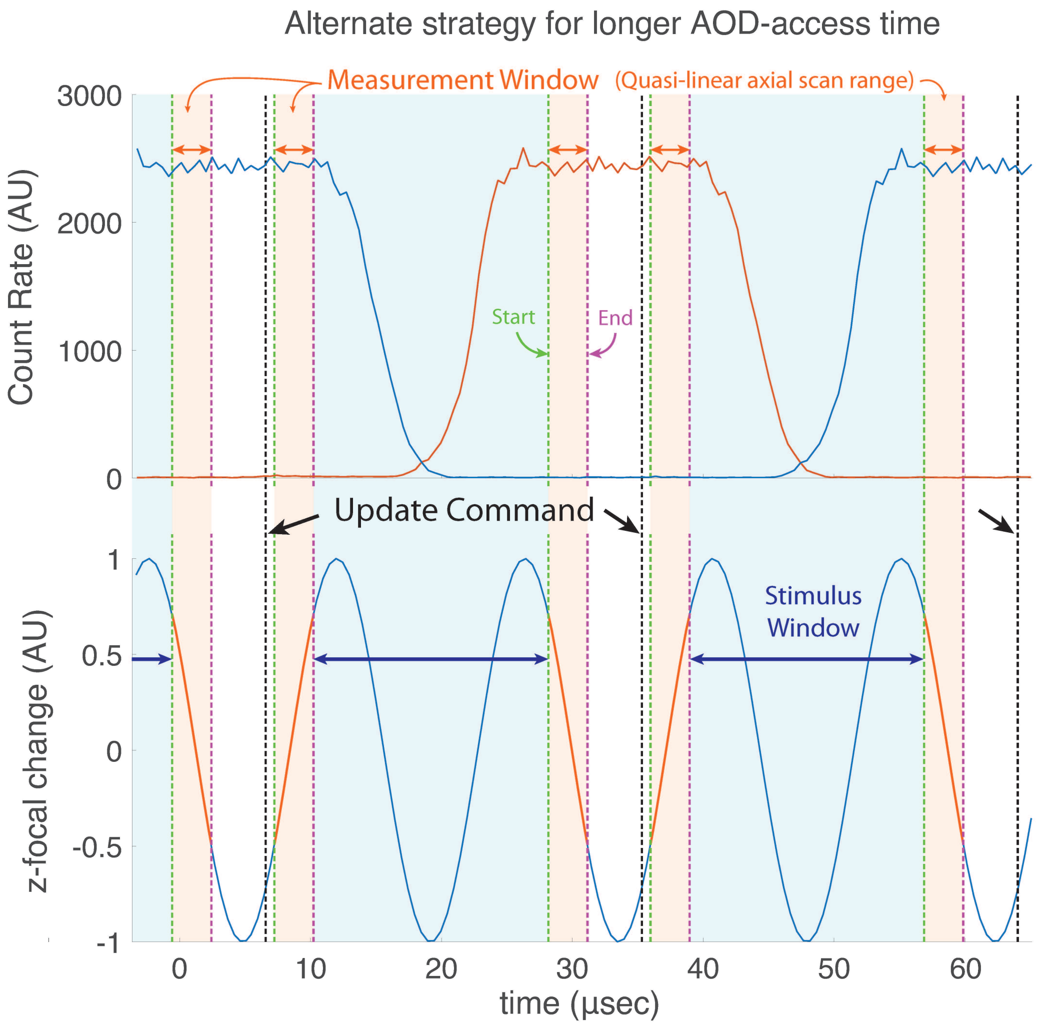

Figure S2. Alternate strategy for longer AOD access time. If the AOD access time is longer than one TAG cycle, we can use 50% duty cycle of 35 kHz (for 70 kHz TAG resonant frequency) and double the access time.

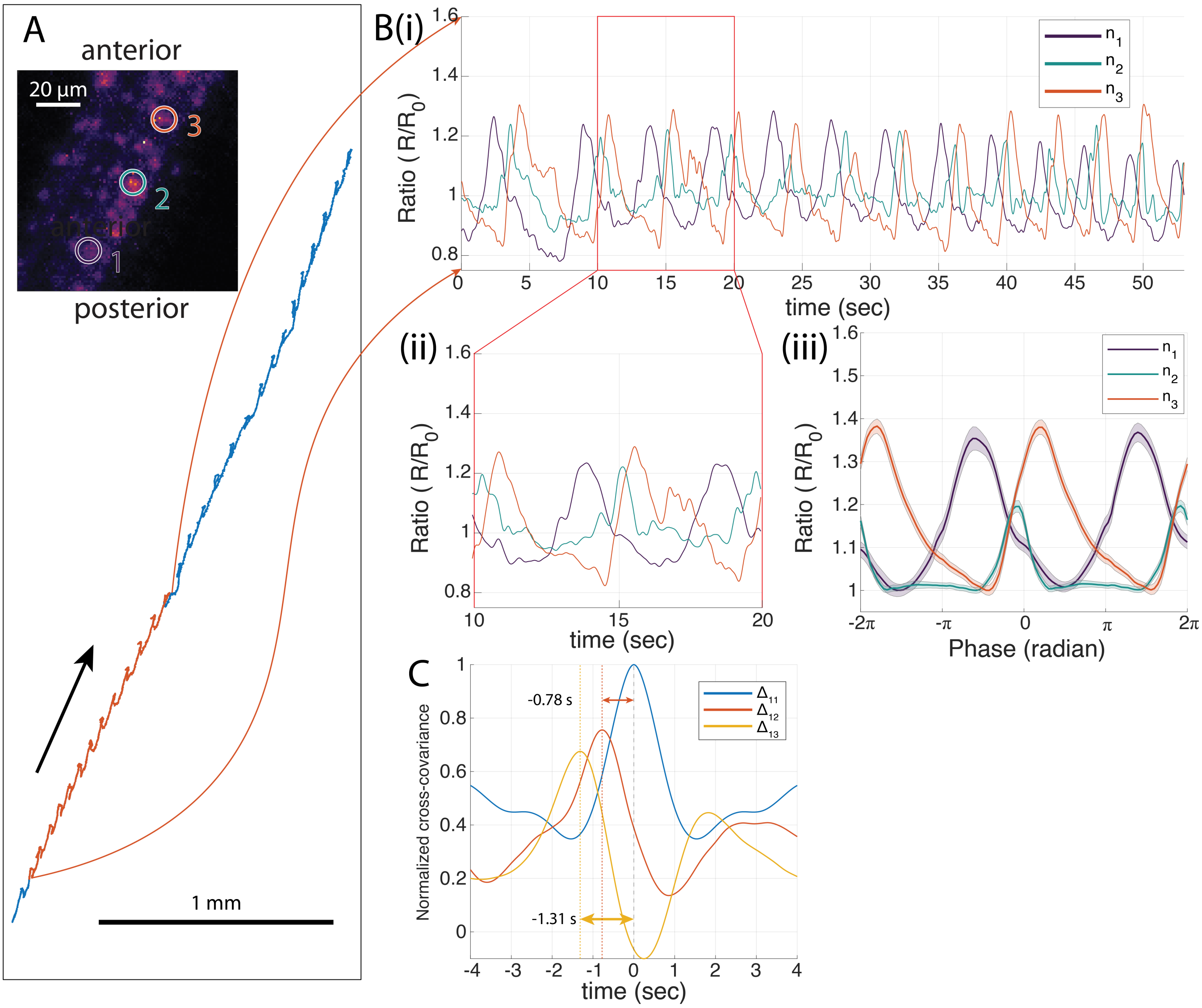

Figure S3. Three-neuron recording of A27h>GCaMP7f;mCherry in a moving larva. (A) The trajectory of one neuron during forward crawling in 97 seconds (blue) with 30 peristaltic cycles. Inset: z-projection of the VNC. The three tracked neurons are indicated by the circles (1-3 from posterior to anterior). (B) ratiometric activity measure (normalized ratio of green to red fluorescence) of each neuron for (i) 55-second (corresponding to the red traces in A) with 13 peristaltic cycles and (ii) 10-second (corresponding to the red box in (i)) excerpts, and (iii) mean neural traces of the 30 peristaltic cycles aligned to the phase of each cycle. (C) Normalized cross-covariance between the ratiometric measure of each neuron and the most posterior neuron. The temporal difference between neurons 1-2, and 1-3 are 0.78 and 1.31 seconds, respectively.

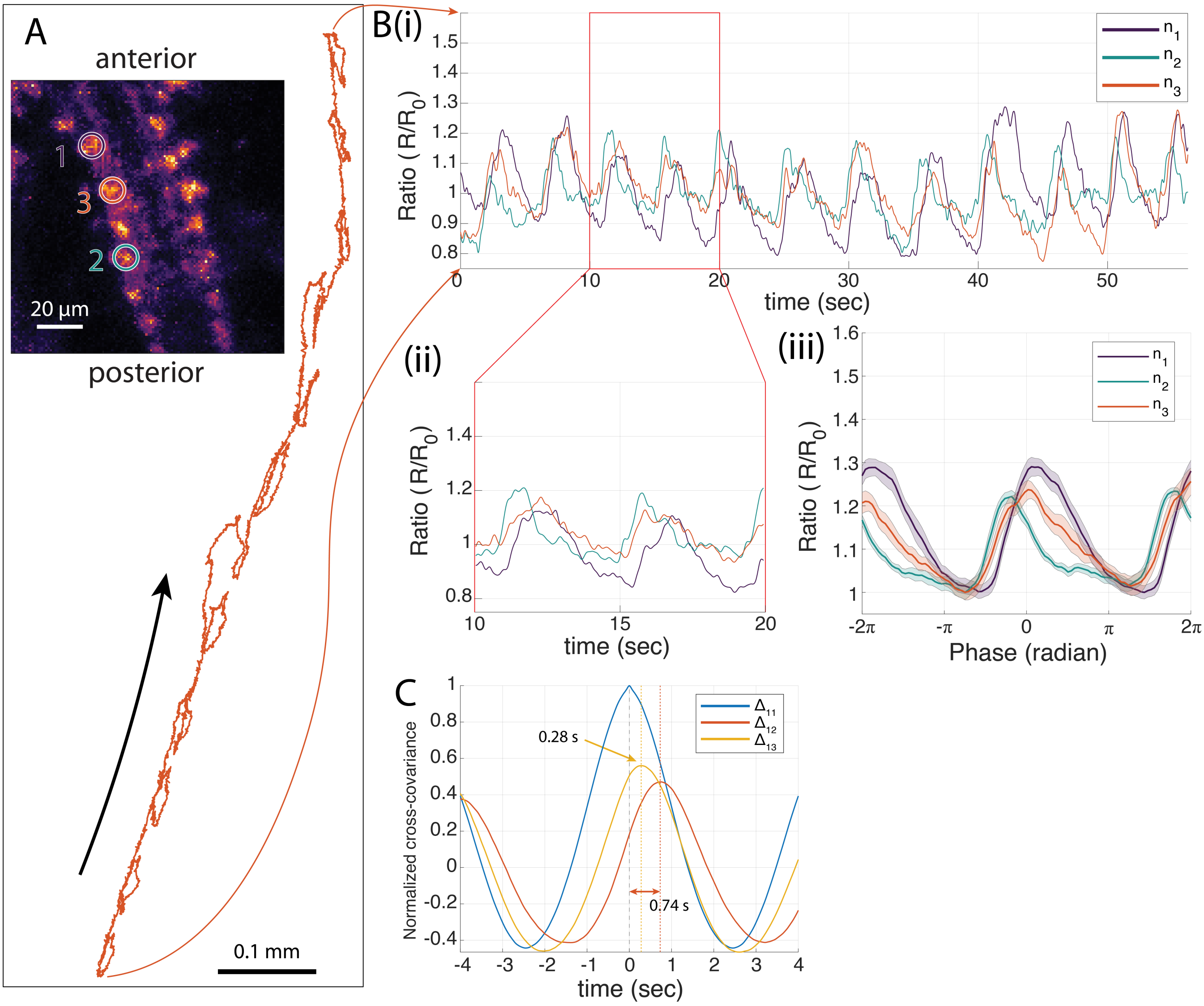

Figure S4. Three-neuron recording of A27h>GCaMP7f;mCherry in a moving larva. (A) The trajectory of one neuron during forward crawling. 12 peristaltic cycles were observed in 55 seconds. Inset: z-projection of the VNC. The three tracked neurons are indicated by the circles (2,3,1 from posterior to anterior). (B) ratiometric activity measure (normalized ratio of green to red fluorescence) of each neuron for (i) 55-second (corresponding to the red traces in A) and (ii) 10-second (corresponding to the red box in (i)) excerpts, and (iii) mean neural traces of 12 peristaltic cycles aligned to the phase of each cycle. (C) Normalized cross-covariance between the ratiometric signals shown in (B.i). The temporal difference between neurons 1-2, and 1-3 are 0.74 and 0.28 seconds, respectively.

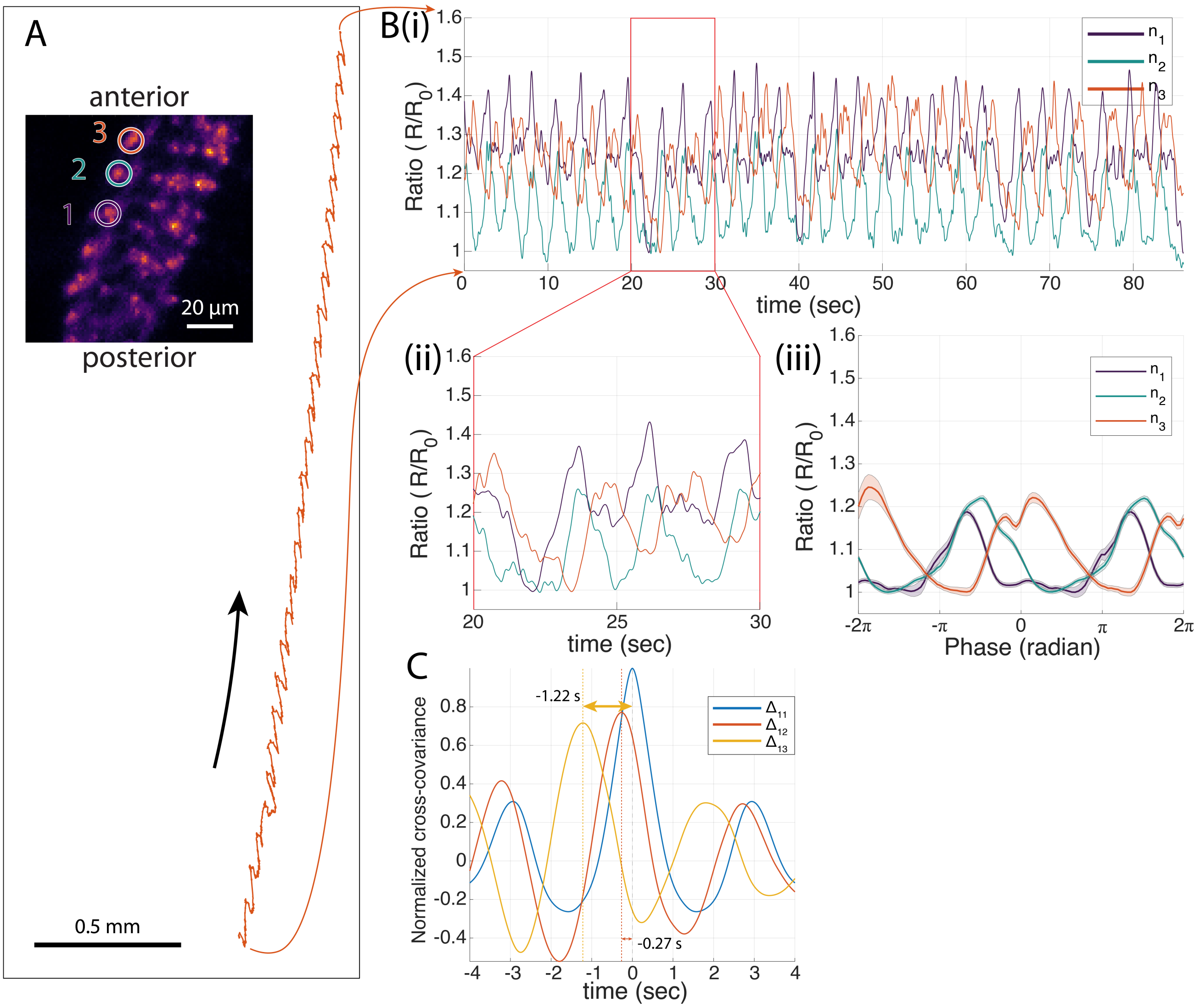

Figure S5. Three-neuron recording of A27h>GCaMP7f;mCherry in a moving larva. (A) The trajectory of one neuron during forward crawling in 86 seconds with 31 peristaltic cycles. Inset: z-projection of the VNC. The three tracked neurons are indicated by colored circles (1-3 from posterior to anterior). (B) ratiometric activity measure (normalized ratio of green to red fluorescence) of each neuron for (i) 86-second (corresponding to the red traces in A) and (ii) 10-second (corresponding to the red box in (i)) excerpts, and (iii) mean neural traces of 31 peristaltic cycles aligned to the phase of each cycle. (C) Normalized cross-covariance between the ratiometric signals shown in (B.i). The temporal difference between neurons 1-2, and 1-3 are 0.27 and 1.22 seconds, respectively.

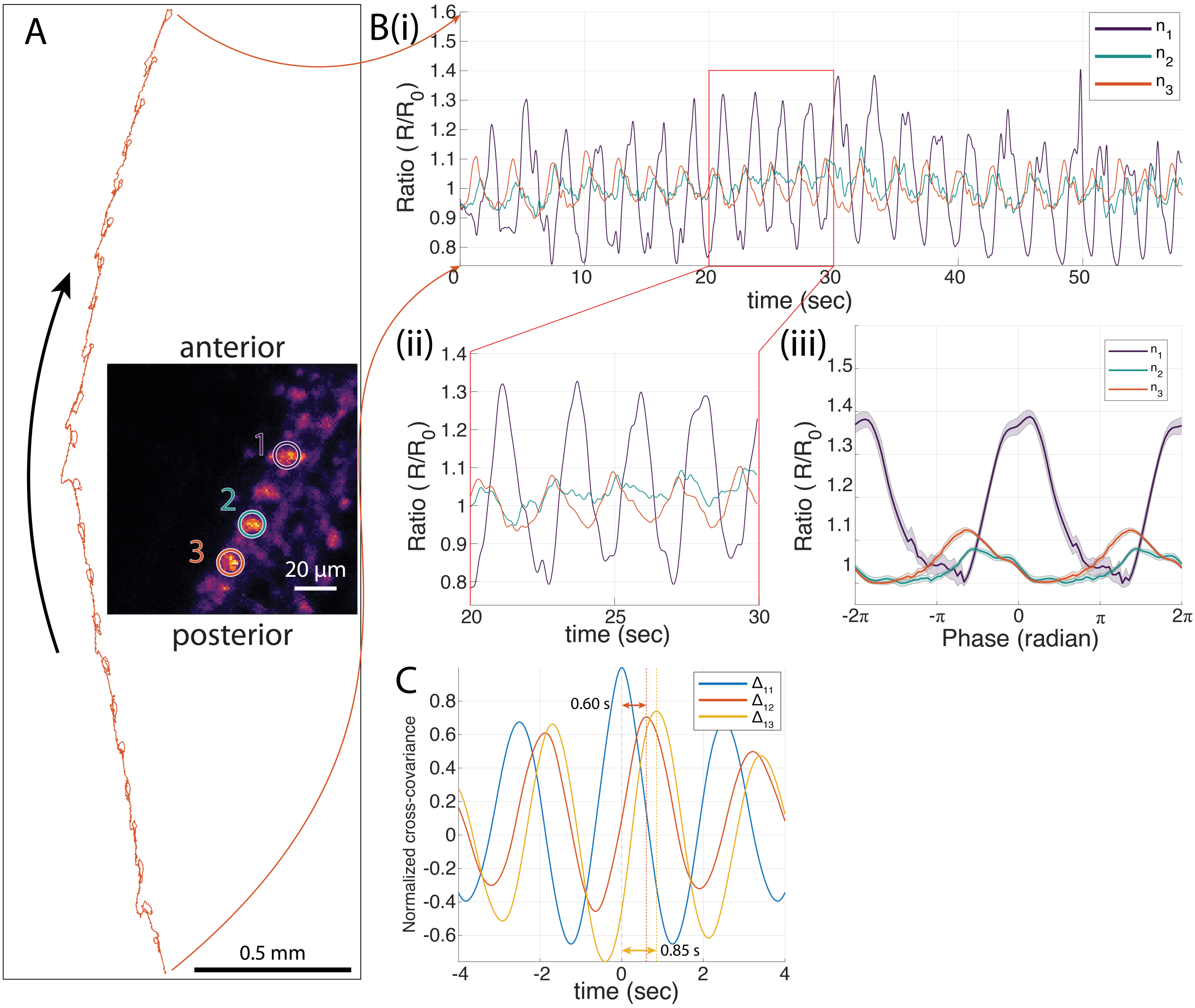

Figure S6. Three-neuron recording of A27h>GCaMP7f;mCherry in a moving larva. (A) The trajectory of one neuron during forward crawling in 58 seconds with 33 peristaltic cycles. Inset: z-projection of the VNC. The three tracked neurons are indicated by the colored circles (3-1 from posterior to anterior). (B) ratiometric activity measure (normalized ratio of green to red fluorescence) of each neuron for (i) 58-second (corresponding to the red traces in A) and (ii) 10-second (corresponding to the red box in (i)) excerpts, and (iii) mean neural traces of 33 peristaltic cycles aligned to the phase of each cycle. (C) Normalized cross-covariance between the ratiometric signals shown in (B.i). The temporal difference between neurons 1-2, and 1-3 are 0.60 and 0.85 seconds, respectively.

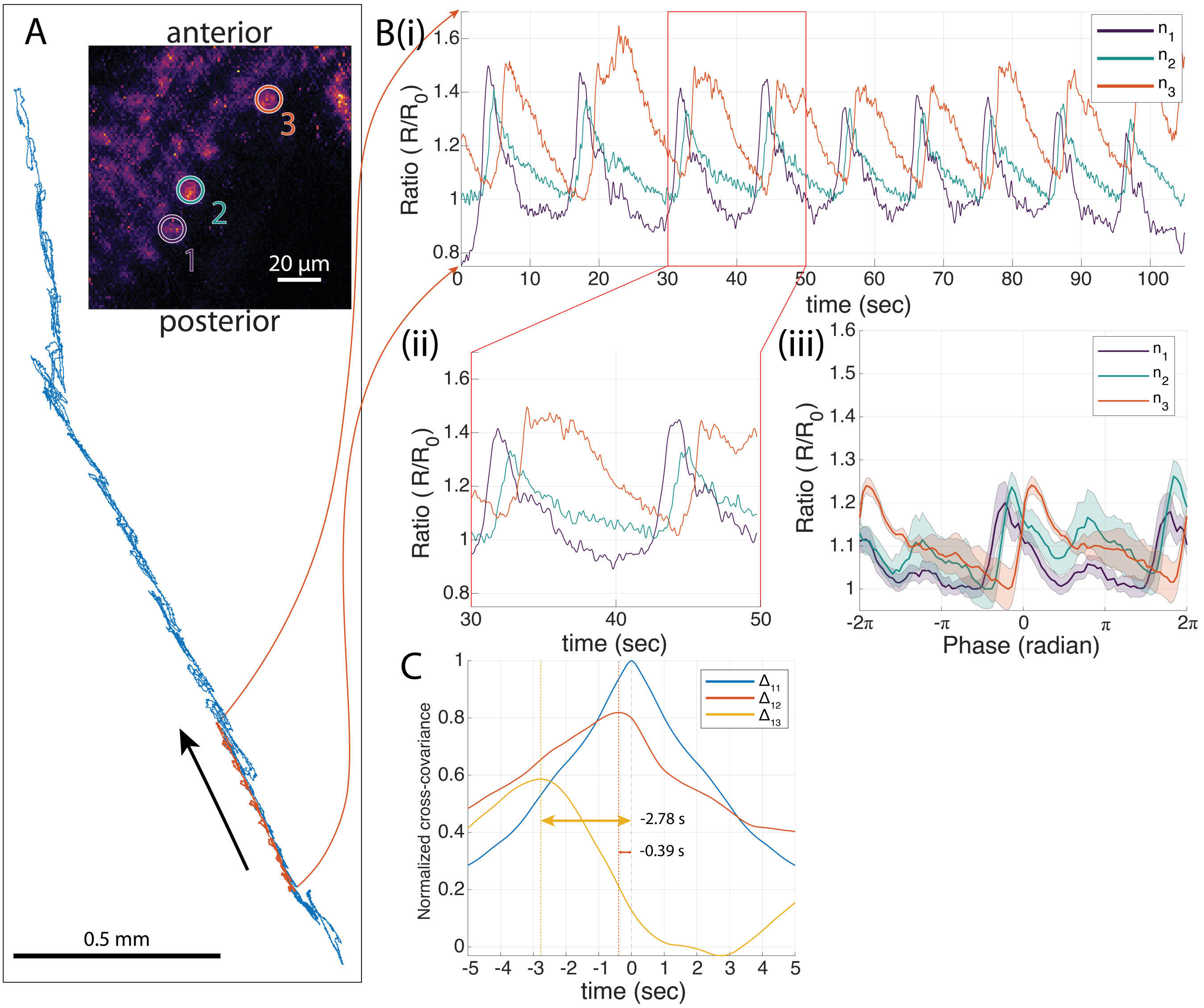

Figure S7. Three-neuron recording of A27h>GCaMP7f;mCherry in a moving larva. (A) The trajectory of one neuron during forward crawling in 183 seconds with 13 peristaltic cycles. Inset: z-projection of the VNC. The three tracked neurons are indicated by the circles (1-3 from posterior to anterior). (B) ratiometric activity measure (normalized ratio of green to red fluorescence) of each neuron for (i) 105-second (corresponding to the red traces in A) with 5 detected peristaltic cycles and (ii) 20-second (corresponding to the red box in (i)) excerpts, and (iii) mean neural traces of the 12 peristaltic cycles aligned to the phase of each cycle. (C) Normalized cross-covariance between the ratiometric signals shown in (B.i). The temporal difference between neurons 1-2, and 1-3 are 0.39 and 2.78 seconds, respectively.

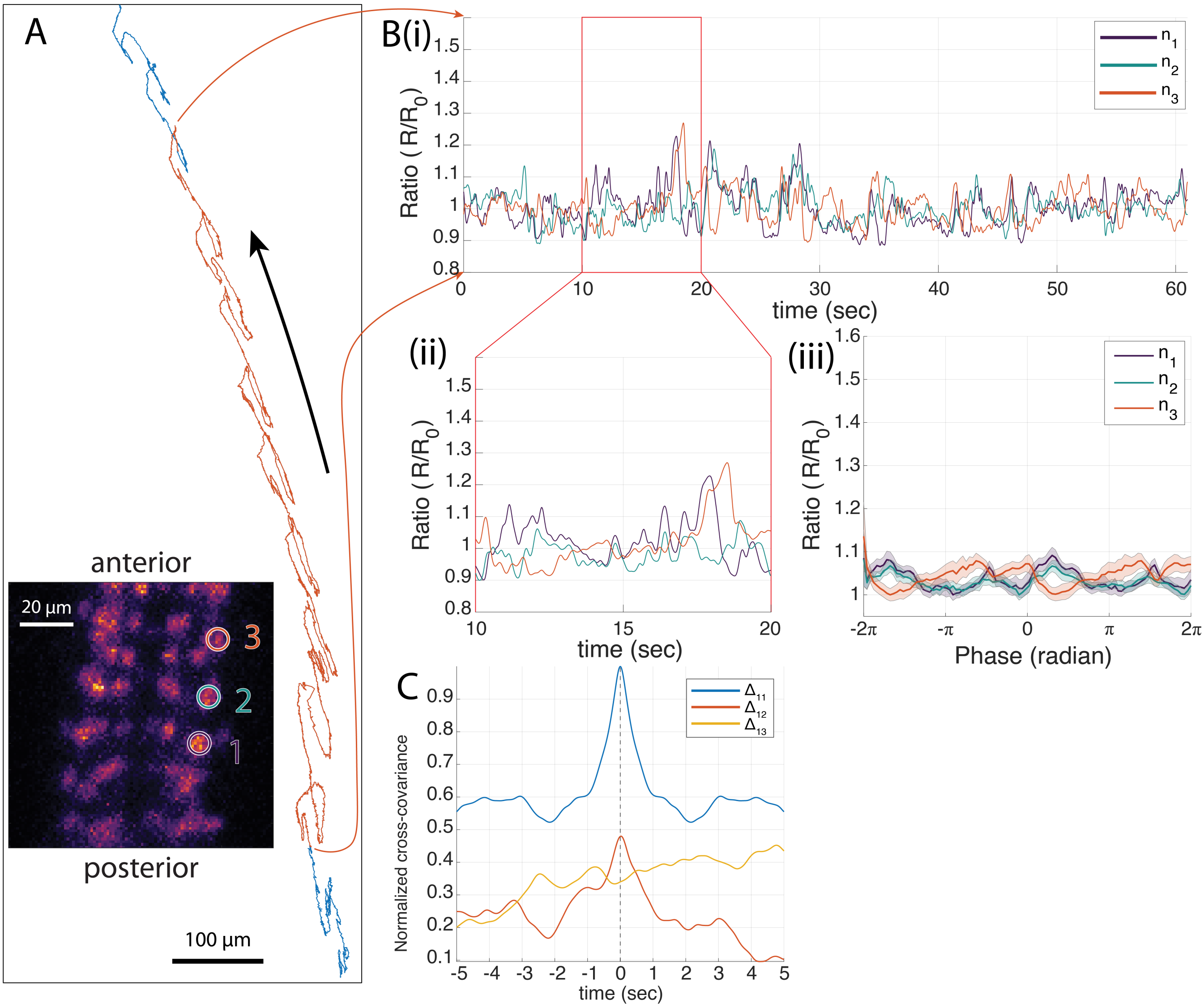

Figure S8. Three-neuron recording of A27h>GFP;mCherry in a moving larva. (A) The trajectory of one neuron during forward crawling in 81 seconds with 18 peristaltic cycles. Inset: z-projection of the VNC. The three tracked neurons are indicated by the circles (1-3 from posterior to anterior). (B) ratiometric activity measure (normalized ratio of green to red fluorescence) of each neuron for (i) 61-second (corresponding to the red traces in A) with 15 peristaltic cycles and (ii) 10-second (corresponding to the red box in (i)) excerpts, and (iii) average traces of 18 peristaltic cycles aligned to the phase of each cycle. (C) Normalized cross-covariance between the ratiometric measure of each neuron and the most posterior neuron. The temporal relation between the signals was not observed.

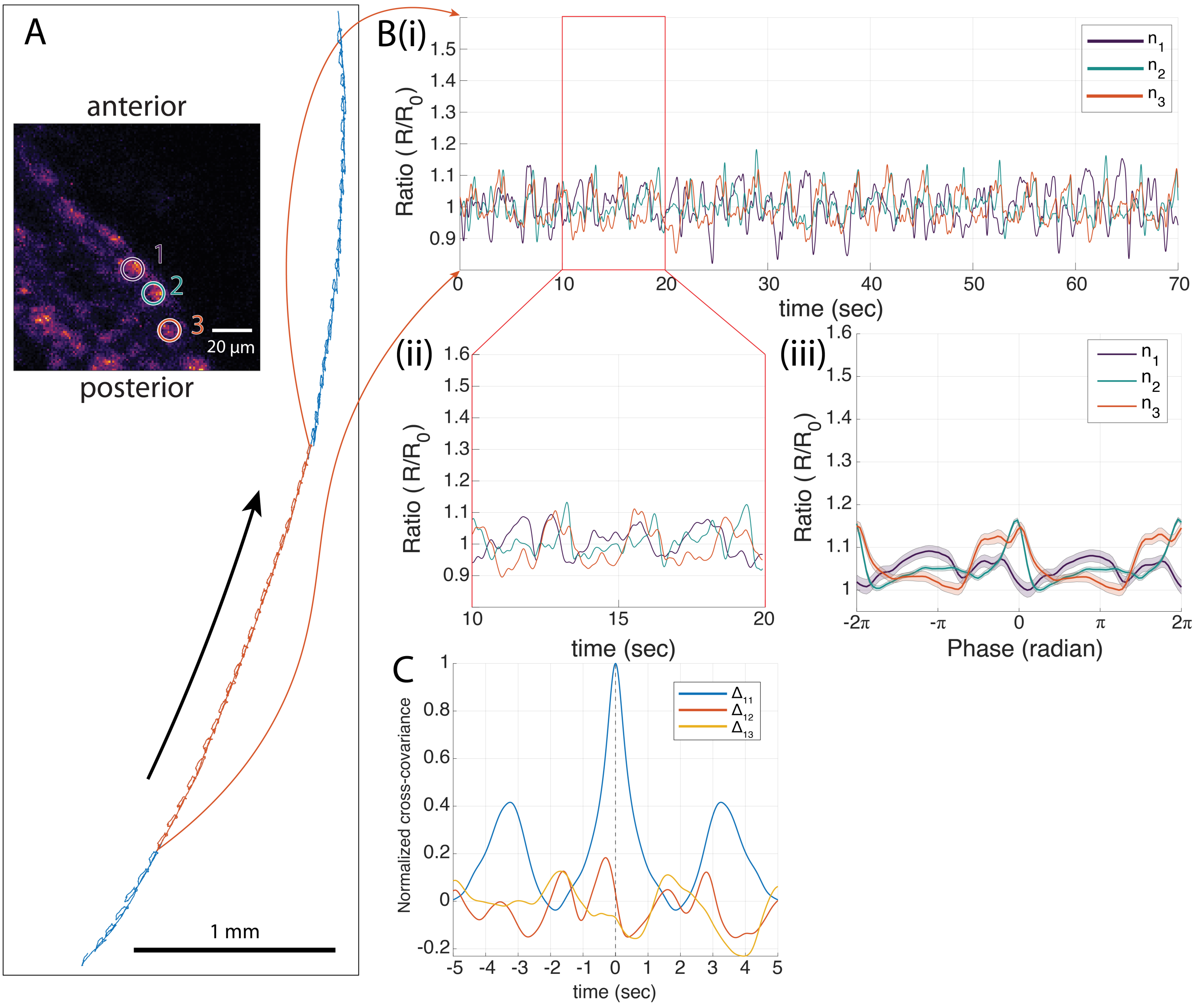

Figure S9. Three-neuron recording of A27h>GFP;mCherry in a moving larva. (A) The trajectory of one neuron during forward crawling in 168 seconds with 48 peristaltic cycles. Inset: z-projection of the VNC. The three tracked neurons are indicated by the circles (3-1 from posterior to anterior). (B) ratiometric activity measure (normalized ratio of green to red fluorescence) of each neuron for (i) 70-second (corresponding to the red traces in A) with 21 peristaltic cycles and (ii) 10-second (corresponding to the red box in (i)) excerpts, and (iii) mean traces of 48 peristaltic cycles aligned to the phase of the cycle. (C) Normalized cross-covariance between the ratiometric measure of each neuron and the most posterior neuron. The cross-covariance between the ratiometric measures is low; the temporal relation between the signals was not observed.

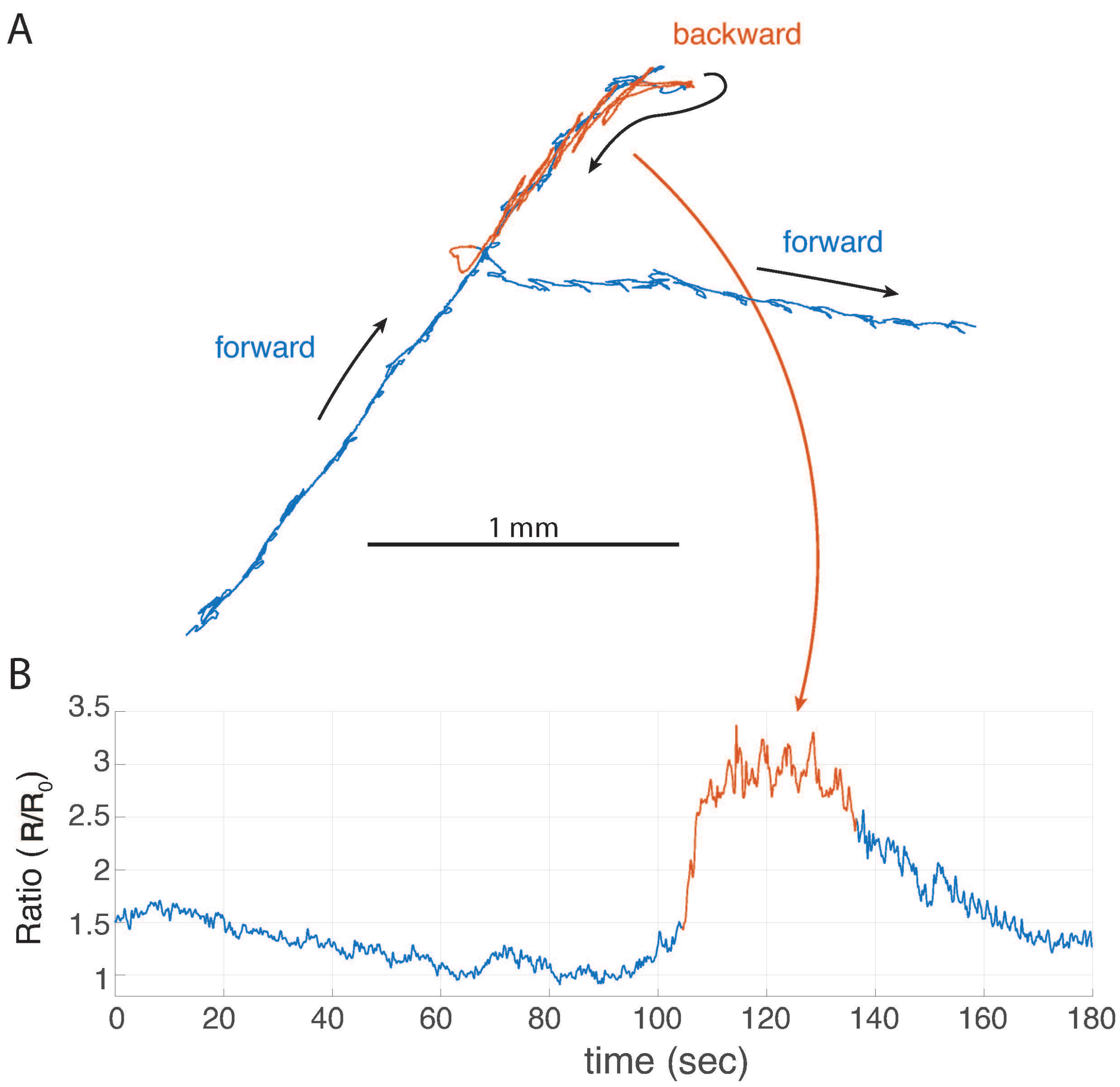

Figure S10. Single neuron recording of MDN>GCaMP7f;Cherry in a moving larva. (A) the trajectory of the tracked MDN. The forward crawling period is shown in blue and the backward crawling period is shown in red. The blue and red indicate the period of forward and backward crawling, respectively. (B) the ratiometric Ca^{2+} activity measure of the MDN during the period shown in (A). The Ca^2+ level is high (the neuron is active) during backward crawling compared to forward crawling.

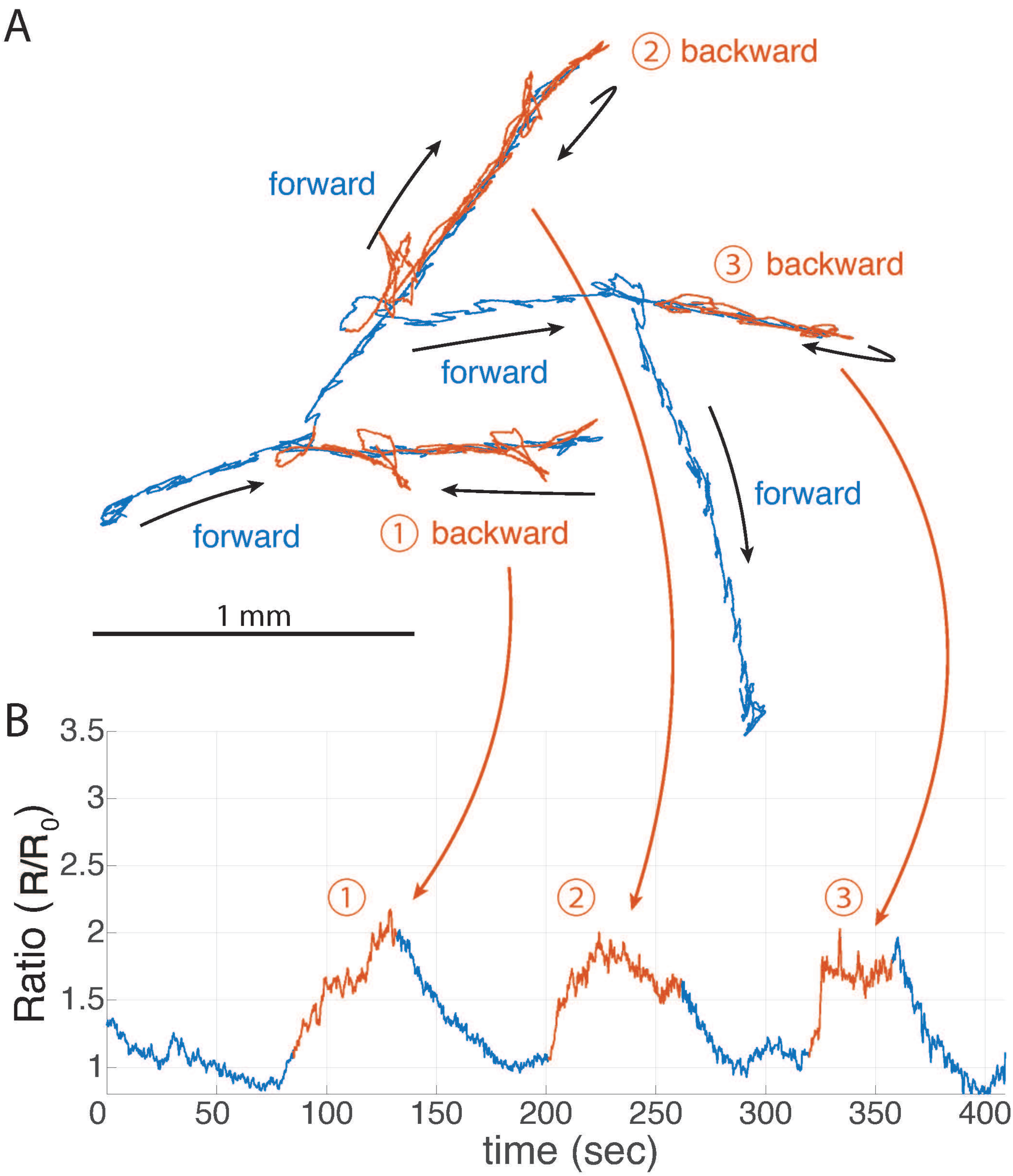

Figure S11. Single neuron recording of MDN>GCaMP7f;Cherry in a moving larva. (A) the trajectory of the tracked MDN. The forward crawling period is shown in blue and the backward crawling period is shown in red. The blue and red indicate the period of forward and backward crawling, respectively. (B) the ratiometric Ca^{2+} activity measure of the MDN during the period shown in (A). The Ca^2+ level increases and remains high (the neuron is active) during backward crawling compared to forward crawling.

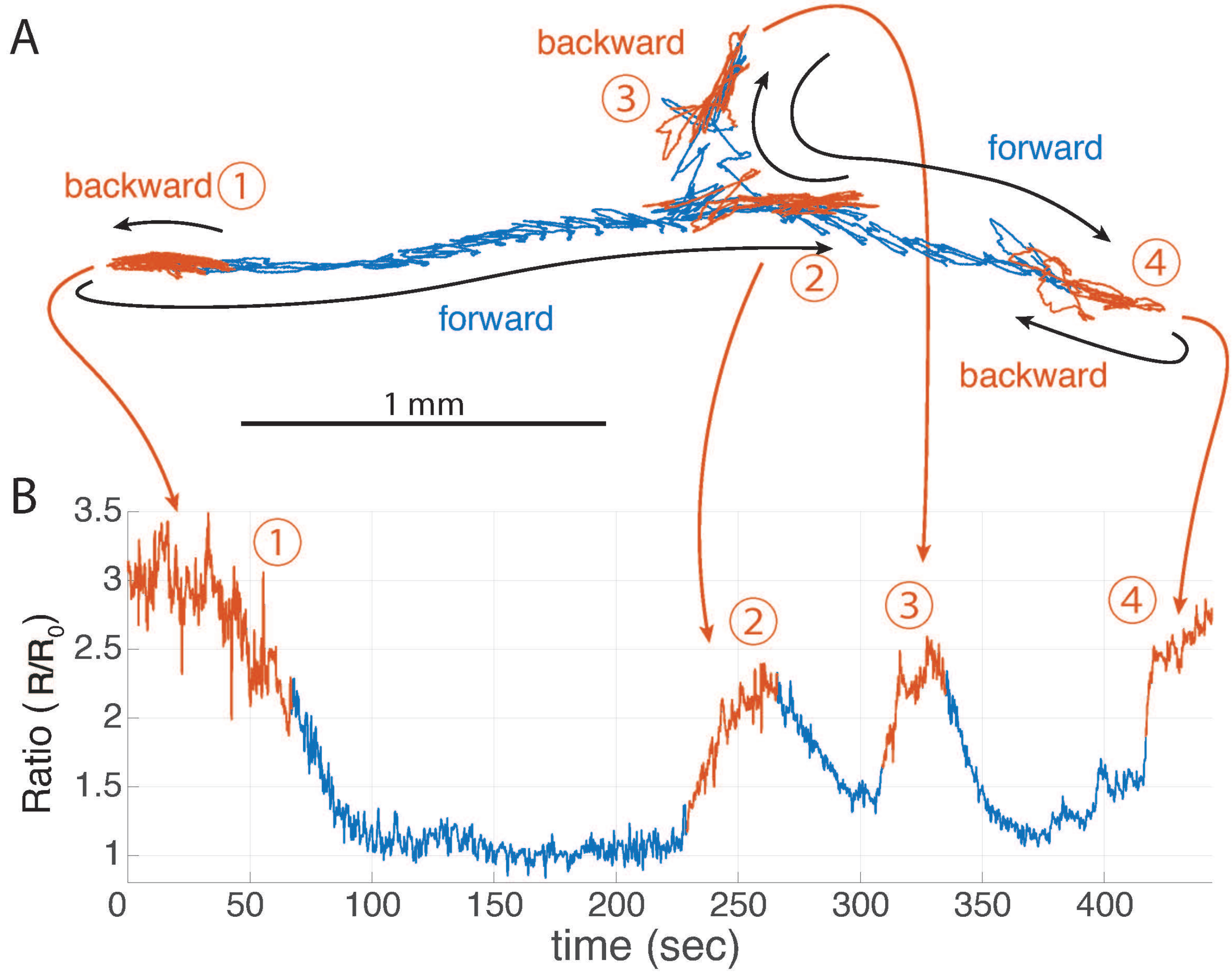

Figure S12. Single neuron recording of MDN>GCaMP7f;Cherry in a moving larva. (A) the trajectory of the tracked MDN. The forward crawling period is shown in blue and the backward crawling period is shown in red. The blue and red indicate the period of forward and backward crawling, respectively. (B) the ratiometric Ca^{2+} activity measure of the MDN during the period shown in (A). The Ca^2+ level is high (the neuron is active) during backward crawling compared to forward crawling.

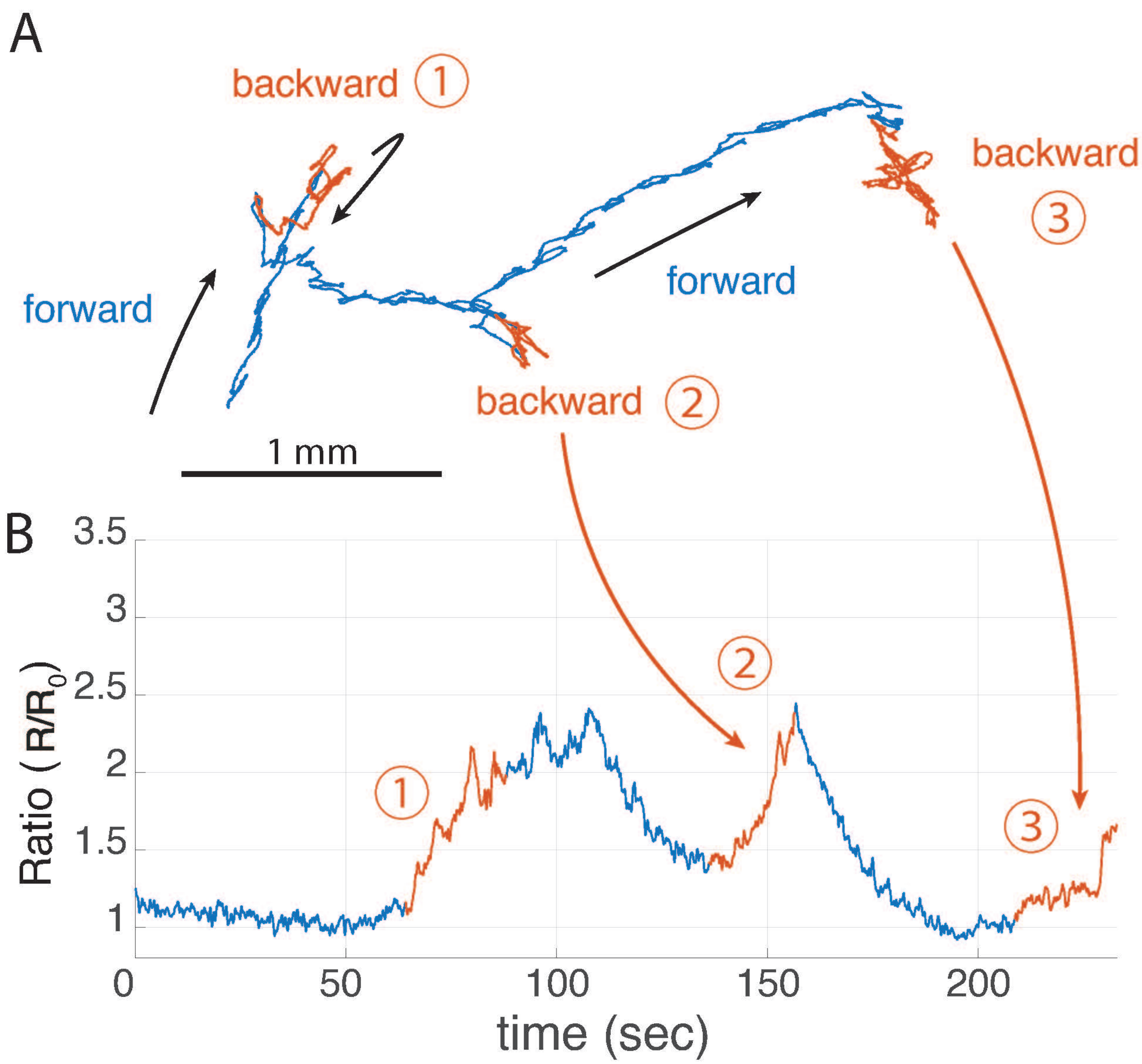

Figure S13. Single neuron recording of MDN>GCaMP7f;Cherry in a moving larva. (A) the trajectory of the tracked MDN. The forward crawling period is shown in blue and the backward crawling period is shown in red. The blue and red indicate the period of forward and backward crawling, respectively. (B) the ratiometric Ca^{2+} activity measure of the MDN during the period shown in (A). The Ca^2+ level is high (the neuron is active) during backward crawling compared to forward crawling.

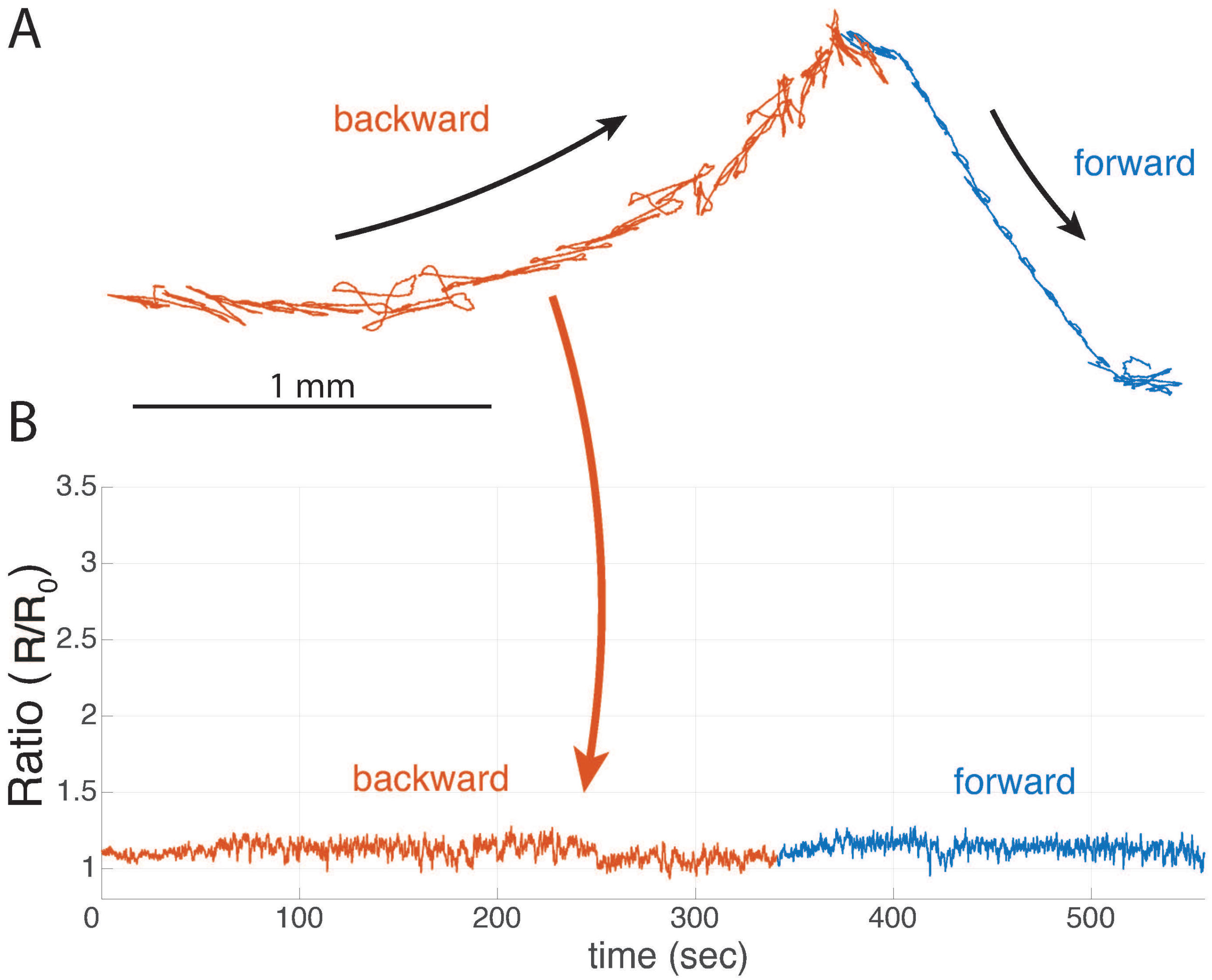

Figure S14. Single neuron recording of MDN>GFP;Cherry in a moving larva. (A) the trajectory of the tracked MDN. The forward crawling period is shown in blue and the backward crawling period is shown in red. The blue and red indicate the period of forward and backward crawling, respectively. (B) the ratiometric measure of the MDN labeled with GFP and mCherry during the period shown in (A). The ratiometric measure is constant throughout the recording period and is independent of the crawling direction.

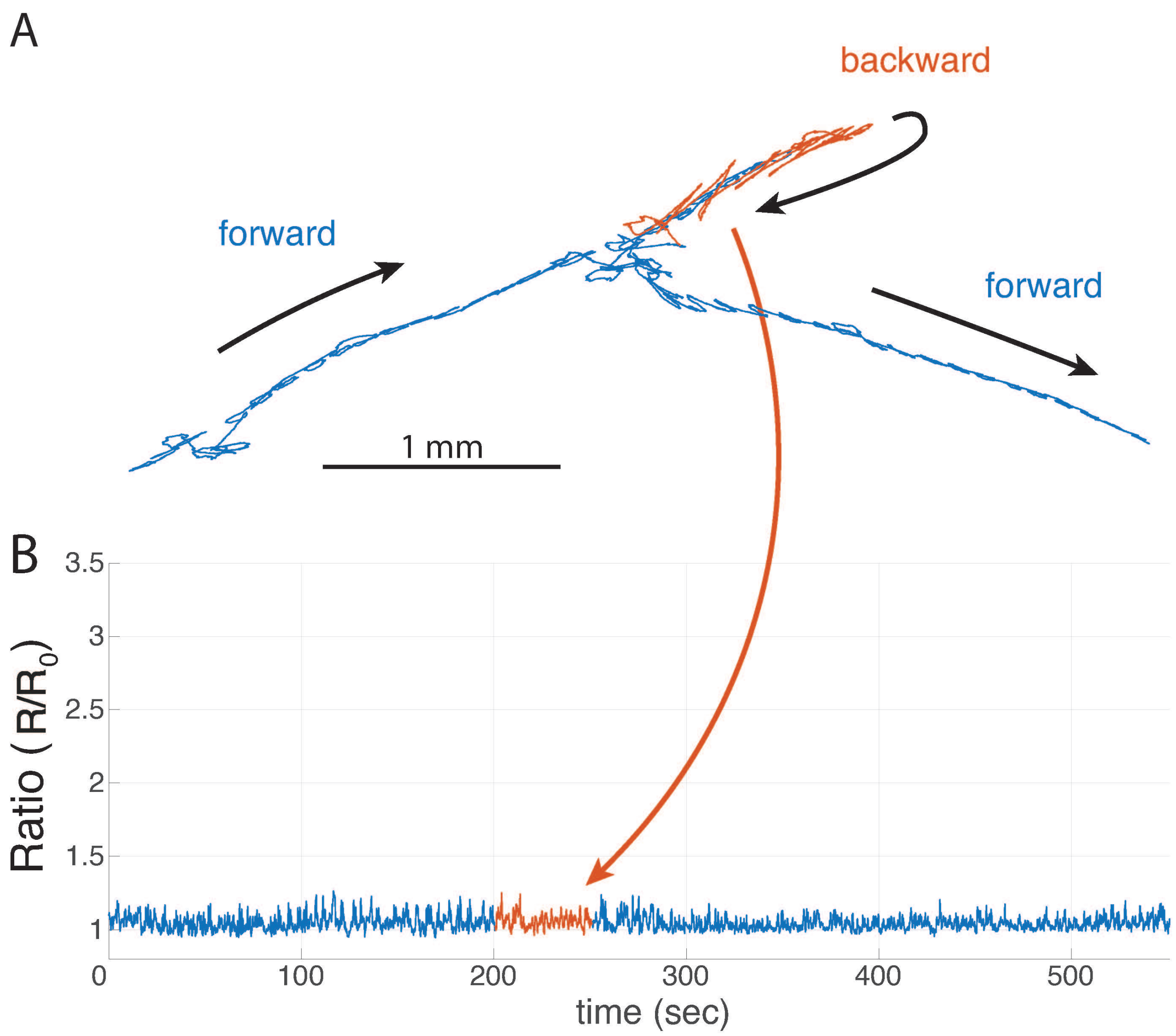

Figure S15. Single neuron recording of MDN>GFP;Cherry in a moving larva. (A) the trajectory of the tracked MDN. The forward crawling period is shown in blue and the backward crawling period is shown in red. The blue and red indicate the period of forward and backward crawling, respectively. (B) the ratiometric measure of the MDN labeled with GFP and mCherry during the period shown in (A). The ratiometric measure is constant throughout the recording period and is independent of the crawling direction.

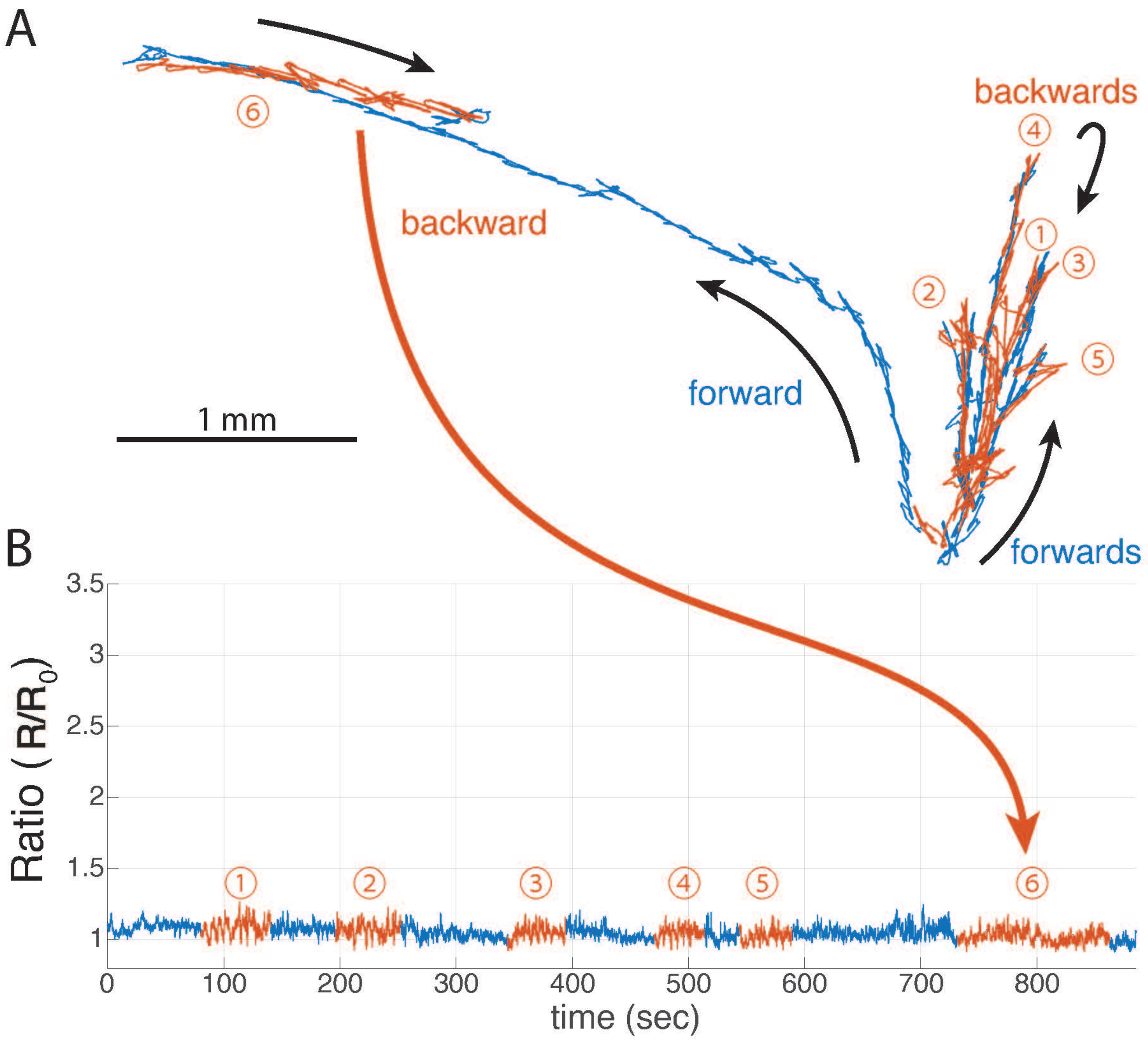

Figure S16. Single neuron recording of MDN>GFP;Cherry in a moving larva. (A) the trajectory of the tracked MDN. The forward crawling period is shown in blue and the backward crawling period is shown in red. The blue and red indicate the period of forward and backward crawling, respectively. (B) the ratiometric measure of the MDN labeled with GFP and mCherry during the period shown in (A). The ratiometric measure is constant throughout the recording period and is independent of the crawling direction.
